# Biological, material and socio-cultural constraints to effective menstrual hygiene management among secondary school students in Tanzania

**DOI:** 10.1371/journal.pgph.0000110

**Published:** 2022-03-14

**Authors:** Dani Stoilova, Rebecca Cai, Sandra Aguilar-Gomez, Naomi Heller Batzer, Elias Charles Nyanza, Anja Benshaul-Tolonen

**Affiliations:** 1 Department of Economics, Barnard College, Columbia University, New York City, NY, United States of America; 2 Columbia University, New York City, NY, United States of America; 3 University of California, San Diego, San Diego, CA, United States of America; 4 Department of Environmental and Occupational Health, Catholic University of Health and Allied Sciences, Mwanza, Tanzania; Tata Institute of Social Sciences, INDIA

## Abstract

Menstrual hygiene management is an important determinant for girls’ educational outcomes. We develop a method of cross-sectional analysis that quantifies the relative importance of four distinct mechanisms: material, biological, social and informational constraints and consider four main schooling outcomes: absenteeism, early departure, concentration and participation. We use survey data from 524 female students enrolled in four co-educational secondary schools in Northern Tanzania. Average age at first period is 14.2 years (standard deviation = 1.1, range 9-19). Information is the least binding constraint: 90-95% of girls report they received information about menstruation and how to manage it. In contrast, biological constraints are hindering: (i) the distribution of menstrual cramps and pain is bifurcated: most girls report very light or very strong pain (rather than moderate) with considerable educational impacts for girls in the latter group, (ii) irregular cycles (62%) and difficulty predicting the cycle (60%) lead to stress and uncertainty. Socio-cultural constraints are binding as 84% would feel shame if male peers knew their menstrual status, and 58% fear being teased over periods. Material constraints include prohibitive costs: girls spending between 12-70% of the daily national poverty line (6,247 TSH per day) on pads during their period. However, we discern no statistically significant relationship between access to pads and absenteeism. In contrast, biological and socio-cultural constraints as well as lack of sanitary infrastructure have significant effects on absenteeism. The results have several implications. First, sanitary pad interventions should consider participation and concentration as main outcomes, in addition to absenteeism. Second, biological (menstrual cramps and pain) and socio-cultural (fear, stigma) constraints are drivers of menstruation-related absenteeism and participation in the classroom and need to be evaluated in trials. We suggest exploring analgesic use, alternative pain-management techniques, menstrual cycle tracking technologies, and social programming in future trials.

## Introduction

Menstrual hygiene management is an important determinant for girls’ educational outcomes in low and middle-income countries. The qualitative research points to a multitude of constraints [1–11] affecting a wide range of outcomes [12–15]. However, the quantitative literature, especially that focusing on impact evaluations, has lagged behind both in terms of the breadth of interventions and the various impacts such interventions can have [13, 16–22]. A few exceptions include a recent feasibility study that took a more holistic approach and included several concurrent treatments such as providing vouchers for analgesics and other pain alleviating techniques as well as provoking community-based discussions about stigma [23]. However, the study design did not allow for identification of isolated channels and its latrine building program did not explicitly target menstrual health management [24].

We contend that prior interventions have focused heavily on material (ex. providing sanitary pads) and informational (ex. amending school curricula) policies on a narrow set of outcomes (enrollment rates or absenteeism). As a result, interventions that are harder to provide and outcomes that are less observable, or require recurring surveying, have been left under-explored [25]. Our study lightly follows in the footsteps of research conducted in Uttar Pradesh by Malhotra et al. (2016) [26] which investigates a variety of factors that may constrain girls’ ability to manage their menstruation, including knowledge and attitudes. The aforementioned study comes to similar conclusions: a majority of girls report being constrained in the management of their menstruation and socio-cultural constraints are common, with many girls feeling isolated or impure during menstruation. While Malhotra et al. (2016) [26] was essential in identifying the constraints faced by girls during menstruation and exploring them through rigorous multivariate analysis, our study aims to highlight the necessity of quantifying the relative impact of various menstruation-related constraints on educational outcomes.

In response, we develop a cross-sectional methodology for quantifying the importance of four distinct aspects of menstrual hygiene management on girls’ educational outcomes. We consider four main outcome variables: absenteeism, early departure from school, concentration, and participation in the classroom. To understand the relative importance of menstrual hygiene constraints on educational outcomes, we utilize our cross-sectional methodology on data from 524 female students enrolled in four co-educational secondary schools in Northern Tanzania. Using data from this context, we quantify these constraints by creating four indices. This allows us to compare their relative contribution to adverse schooling outcomes through regression analysis.

The paper makes four contributions to the literature. First, we collect and separate reasons for menstruation-related absenteeism and lowered classroom participation. Second, we explore girls’ stated reasons for absenteeism and changes in concentration and participation in the classroom to determine the most binding constraints. Third, we develop a cross-sectional methodology meant to operationalize each constraint type, quantify the relative importance of each menstruation-related constraint and to guide future research. Lastly, we increase our understanding of how socio-cultural constraints—including restrictions at home and fear of harassment, shaming, and sexualization from peers and teachers—affect schooling outcomes by privately surveying a large number of girls yielding unique prevalence estimates of period teasing and harassment.

## Background

Menstruation has been found to pose a threat to girls’ educations around the world [1, 25]. The MHM in Ten Agenda [2], directly investigates the relationship between menstruation and education in an effort to make education more equitable for female students and, simultaneously, improve young women’s access to schooling. Our study aligns with the MHM in Ten Agenda by presenting additional cross-sectional research on menstruation-related constraints to aid in the development of global MHM guidance.

The extant literature emphasizes the effect of menstruation on girls’ educational outcomes, but the bulk of previous impact evaluation research focuses solely on absenteeism and enrollment rates. Several reviews covering over a hundred individual studies share a common prevalent finding; across the LMIC, girls self-report higher rates of absence while menstruating compared to when not menstruating (e.g. reviewed in Southern and East Africa [6]). However, in addition to absenteeism, research from Sub-Saharan Africa [14, 27, 28] has found that girls self-report worsened school performance, decreased mobility, and difficulty concentrating during their period. As a result, we investigate three gaps in the measurement of educational outcomes.

First, we identify only one study [23], to date, that has quantified how often girls leave school early (end their school day prematurely) because of their period. Although leaving early is not often measured, missing parts of a school day can have a significant impact on the quality and continuity of education if the behavior persists. Second, additional educational outcomes, in particular girls’ participation and concentration in school are under-studied. Although participation and concentration are integral in developing a comprehensive picture of girls’ educational attainment, few studies, thus far, (e.g. [14, 29]) quantify menstruation’s effects on these subjective outcomes. Third, very few quantitative studies, to date, have examined the *reasons* behind absenteeism and decreased participation (e.g. [30, 31]), though they have been explored qualitatively [14]. Although girls’ self-reported reasons for absenteeism may not capture community level determinants of educational disparity, this research is crucial for the development of future interventions that target individual outcomes.

Previous literature has loosely identified various interrelated but conceptually distinct types of constraints associated with menstruation. For instance, a recent study was able to measure girls’ confidence in undertaking menstrual management at school and in the home. Similar to our paper, the study found that various personal, interpersonal, and environmental factors contribute to each of the contexts indicating that multiple elements need to be considered and there is no single solution to improving the menstrual experience [22]. Based on this, we utilize four menstruation-related constraint types to utilize throughout our analysis: informational, socio-cultural, biological, and material. Individually, each component has been empirically shown to harm girls’ mental health, their ability to hygienically manage their period, and their likelihood to remain in school and succeed academically. These constraints share many similar consequences, but each has different causes and, thus, distinct policy solutions. We acknowledge that each constraint type does not exist independent from the others, therefore, we develop a methodology that combines and considers the impact of each constraint on educational outcomes.

### Informational constraints

Often, girls may receive insufficient, inaccurate, or incomplete educations about the physiological process of menstruation and the hygienic management it requires. Much of the early literature on this topic focuses on the high number of girls who start menstruating without being informed why menstruation occurs or how to manage it [7, 28, 32]. For example, a survey of 25 districts in five countries in southeastern Sub-Saharan Africa estimates that 66% of girls did not know about menstruation before menarche [5]. In Ethiopia, a previous study found that 49% of girls did not learn about menstrual management before menarche [14]. Similarly, in the Kilimanjaro district of Tanzania many girls transition through puberty without adequate guidance on puberty or menses management [12].

Menarche without prior information is commonly associated with negative emotional reactions, including shock, fear, and lack of confidence to seek help [4]. Other risks of insufficient information may include unintended pregnancy and subsequent marriage [6] and urogenital infection [33].

While some interventions have incorporated puberty and menstrual education [34–36], few have examined the effect of such education on attendance. Examples include a non-randomized trial among 120 schoolgirls in Ghana whcih found that education-only or education-plus-pads increased girls’ attendance by the same amount, roughly 9%. In addition, the Nia Project, an ongoing large-scale intervention of 140 primary schools in Kenya, intends to evaluate the effects of puberty education, sanitary pad provision, and a combination of the two on school attendance, among a wide variety of outcomes [16, 37].

### Socio-cultural constraints

We consider socio-cultural constraints as norms or interpersonal interactions within communities that can prevent girls from attending school or negatively affect their educational experience. A growing body of evidence suggests that girls commonly report feelings of embarrassment and shame related to menstruation, often these emotions are borne from both internalized and externally imposed constraints [1, 4, 7, 38]. These findings confirm the significant impact of menstruation-related socio-cultural constraints on girls’ schooling and educational outcomes.

We identify three interrelated but discrete phenomena in the literature: teasing, restrictions on menstruating women, and sexualization. First, in-school period teasing may cause girls to feel shame or discomfort, which, as a result can deteriorate girls’ mental well-being or compel them to avoid attending school. We cover period teasing extensively in Benshaul-Tolonen et al. (2020) [39]. Second, a multitude of qualitative studies have documented the existence of explicit cultural or religious restrictions on menstruating women [1, 40, 41]. For instance, a systematic review from Sub-Saharan Africa finds consistent evidence that menstruation is commonly associated with potential contamination, leading to restrictions on women cooking, touching water or livestock, and participating in community/religious gatherings [42]. We further explore the prevalence of restrictions in the Tanzanian case study in Benshaul-Tolonen et al. (2020) [39]. Third, members of the community may commonly associate menarche with sexual maturity, marriageability, and childbearing; thus menarche may be a determinant of premature school dropout. Several case studies [13, 43] in Sub-Saharan Africa and a review of the LMIC [44] find that early menarche is associated with early sexual debut, early marriage, pregnancy, and increased risk of sexually transmitted infections. Qualitative work in Tanzania has documented instances in which post-menarche girls are sexually propositioned or offered money for sex. As a result, these instances may lead to anxieties about sexual harassment and pregnancy or, in some circumstances, dropout [4, 12]. The prevalence of these instances and fears, however, is unknown.

Compared to the other constraint types discussed throughout this paper, there is a dearth of quantitative evaluations focused on socio-cultural interventions. However, a growing number of authors recognize the benefit of investigating socio-cultural interventions. Preliminary studies have investigated the importance of including boys and men, family, and communities in education to combat stigmas and taboos [7, 32, 39].

### Biological constraints

A developing branch of menstruation-related literature has brought to light the significant impact of biological constraints on young women’s educational outcomes. Biological constraints can range from dysmenorrhea (menstrual cramps) to more severe disorders like endometriosis. In particular, dysmennorhea is a common gynecological complaint among adolescents in developing countries [8, 29]. A systemic review of 50 studies [45], and various single case studies [15, 31, 46], have reinforced the finding that cramps are a common cause of school absenteeism and reduced participation. From this, we are able to conclude that menstrual pain, without proper diagnosis and management, can prevent girls from attending school and inhibit their ability to focus or participate in class.

Educational interventions and the provision of pain management medication can be used to address biological constraints. However, no study, to date, has evaluated pain management as an isolated treatment. A multi-component pilot intervention among 232 girls in Uganda trained teachers in pain management strategies (stretching, using a hot-water bottle) and provided analgesics (painkillers) alongside other interventions [23]. After 9 months of treatment, the study found evidence that the proportion of girls using painkillers and those using at least one pain relief method increased as a result of the intervention [23].

### Material constraints

Menstruation-related material constraints often fall into two categories: i) access to adequate menstrual hygiene management absorbents and ii) access to satisfactory WASH facilities––infrastructure that includes water, soap, privacy, and space to change. Correspondingly, a study completed in the Kamuli district of Uganda found that 90.5% of secondary school girls did not meet the criteria for adequate menstrual hygiene management [47].

The use of unsanitary and sub-standard menstrual absorbents is commonly observed, but is particularly prevalent among girls of low socio-economic class or in rural areas [8]. Documented accessibility issues include high prices for hygienic materials, little or no availability in local areas, or interpersonal deterrents from buying, such as embarrassment buying from male store owners [48]. As a result, in the southeastern African region, including Tanzania, common absorbent materials include natural materials (grass, leaves, cow dung, tree bark), cotton, animal skins, rags, and mattress stuffing [5]. However, if girls are provided with MHM absorbents such as pads or menstrual cups and proper support, they are likely to utilize the methods [17, 49].

Access to WASH facilities is a critical determinant of girls’ menstrual hygiene management since the body and reusable absorbents must be washed several times a day and then dried to meet adequate MHM standards according to current guidelines [3]. A case study from Sierra Leone found that girls without access to adequate WASH facilities during the school day may be forced to go home early, improperly dispose of their absorbents, or use latrines outside of school grounds leading to disruptions in their education [50].

The primary concern is that girls experiencing material constraints are at higher risk of infection, leaking, and smells. Not only does this pose a threat to girls’ physical health, but it has been found to encourage teasing behaviors from classmates [39]. Additional research from Odisha, India, establishes a link between women’s experiences with sanitation and their mental health [51]. Another, distinct area of concern is that girls may engage in transactional relationships or sex in exchange for pads and other unaffordable MHM materials [6, 9, 52]. Combined, these factors emphasize the importance of addressing menstruation-related material constraints to improve girls’ well-being and educational outcomes.

Accordingly, material policy solutions can also be broken down into two categories: the provision of sanitary materials and the improvement of WASH facilities. There is cross-sectional evidence suggesting that girls who use pads are less likely to be absent during their period [14, 31], but these studies fail to account for probable omitted variables, namely household income, parental education, and knowledge about menstruation. In addition, there are few randomized program evaluations that address a causal link between pad use and attendance or participation in school. Within the Sub-Saharan African context, a pilot non-randomized intervention in Ghana [53] and a quasi-randomized intervention in Uganda [21] found that girls in sanitary pad treatment groups had improved attendance, but they were unable to establish causality. A larger cluster randomized study from Kenya followed 644 girls over 10 months and found no evidence, or weak evidence, of reductions in absenteeism due to a menstrual cup and sanitary pad program [18, 37]. In Nepal, girls who randomly received menstrual cups and sanitary pads were no less likely to miss school during their period [19]. In addition, the provision of disposable sanitary pads can have a relatively low return on investment due to high unit cost, compared to reusable sanitary products such as menstrual cups [54], therefore, it is essential to determine the intervention’s relative efficacy in improving educational outcomes.

On the other hand, the study of WASH facilities on menstruation-related absenteeism is less developed and remains largely observational and qualitative (e.g. [15]). A notable exception is a cluster-randomized trial of roughly 6,000 students in Kenya. Studies on the intervention found that improvements in latrine infrastructure and cleaning reduced girls’ absenteeism, whereas latrine cleaning and hand-washing campaigns alone did not significantly affect attendance [24, 55].

## Materials and methods

### Sampling

The sampled students were enrolled at four co-educational secondary schools in the Sengerema District (Mwanza Region) and Geita District (Geita Region), Tanzania. The bulk of the schools were rural, but one school was peri-urban. Three of the schools were day-schools, while one had a girls-only hostel. While the selection of four schools is not representative of the regions or of Tanzania as a whole, the in-school sample is representative due to randomization. The study used a random sample of all students in the target secondary school grades (1–3). Systematic sampling, within the schools, was done using the class registrar. Every third student on the registrar was selected to participate, provided they were present at the school. In total, 432 male and 524 female students participated in the study. Sample size was limited to 524 female students and four schools because including more schools and individuals was cost-prohibitive at the time of program development. Results from the boys’ surveys, and selected results from the girls’ surveys are published in Benshaul-Tolonen et al 2020 [39].

We undertook data collection in May of 2019 with one male Tanzanian lecturer (co-PI), two female US-based scholars, and two local research assistants (one male, one female). All research staff were present in the classrooms during surveying, providing a mixed-gender research group. Classroom teachers helped hand out and collect the surveys as well as keep order in the classrooms.

To avoid congestion of students in classrooms, special classrooms were assigned for the study––up to six rooms per school. Although boys were included, they were separated from the girls during surveying. We determined beforehand that a pen and paper survey would allow students the maximum amount of privacy and increase the likelihood of honest answers as compared to an enumerator-led survey. All female students received a fabric gift bag with a notepad, a pen, and a menstrual health pamphlet from the NGO AFRIpads upon completion of the survey and, at a later date, a set of three reusable sanitary pads.

The survey contained a knowledge assessment section, questions about menstruation-related experiences, attitudes, information sources, and teasing behaviors. The original Swahili questionnaires with English translations are included in the supplementary information section. The questionnaires were not validated on a student population prior to use since the study was considered a pilot study. In addition, field researchers made qualitative observations on each school’s latrine infrastructure.

### Measurement of outcomes

A review of nationally representative data from 12 African countries, Loaiza and Lloyd (2008) found similar rates of absence between boys and girls [56], further corroborated by research in Kenya [37]. This may imply that girls partly miss school due to menstruation, but boys miss school at the same rate for separate reasons. In this study, we will measure absenteeism as days missed during a girl’s last period, rather than overall rates.

Four distinct educational outcomes are considered in this study: absenteeism, leaving school early, participation in the classroom, and concentration in the classroom. The survey asked students to recall their behavior during their last menstrual period. Specifically, each outcome behavior was linked directly to menstruation; e.g. “During your last period, were there days you could not attend school *because of your period?*”. In this study, we decided to separate absenteeism into two distinct outcomes: missing school entirely and leaving early. This decision was made as we believe that the two variables may have different effects, but also different origins. For instance, while absenteeism may be more likely due to feeling unprepared, leaving early may be more closely linked to being unable to manage your period during the school day which may result from unexpected timing of one’s period or inadequate materials and WASH facilities. However, it is possible that responses suffer from both recall bias and social desirability bias. Nevertheless, we highlight three strengths to our approach.

First, the paper’s primary strength is its unique quantitative data on *reasons* for absenteeism and other behaviors. Girls were asked to report why they had engaged in each of the four behaviors, choosing multiple answers from an extensive list. Second, we glean insight into the *relative prevalence* of different educational barriers. Third, by asking girls to report absenteeism or early departure caused directly by their period, we reduce risk of capturing absenteeism that is merely coincident with, rather than caused by, their periods.

### Ethical considerations

Research permits for the study were received at the Bugando Medical Centre and Catholic University of Health and Allied Science Joint Ethics and Review Committee, and National Institute for Medical Research (NIMR) (Ref: MR/53/100/596) in Tanzania. IRB was approved at Barnard College, Columbia University (Ref: 1819–1110-010) in the USA. Permission to conduct the research were obtained from the Tanzania Commission for Research and Technology (COSTECH) and at Geita District Council (Ref: GDC/E.10/1/VOL.2/267).

The survey was conducted in four selected schools in the Senegrema and Geita Districts. Permission was obtained from district secondary school education officers and medical officers of health in order to collect data in the secondary schools. Likewise, written consent was obtained from each school’s headmaster or headmistress for their school’s participation in the study.

In addition, the students signed written assent forms in Kiswahili (the primary language of most of the population of Tanzania) prior to data collection. The forms explained the purpose, risks, and significance of the study, as well as participants’ right to participate or withdraw. Students’ parents were informed about research activities through their school management committees; however, written consent was not required from the parents according to the local ethics reviews.

### Statistical analysis

All statistical analysis was performed in Stata v.15.1. To obtain the regression coefficients in Fig 4, we use a linear probability model. The model reports correlations between social, biological, and material constraints with the four educational outcomes of interest (absenteeism, leaving early, participating, and concentrating less). This identification controls for but does not report coefficients on respondent’s age, grade, and age at menarche, as well as school fixed effects.

To develop the three constraint indices, the 2–3 most applicable questions to each constraint were selected, scored, and then normalized to fall between zero and one. For each constraint type, individual students’ points are added together and then divided by the maximum amount of points to find the normalized score. The constraint indices were developed post-hoc, therefore, we are limited in our interpretation as they were not developed a-priori. Further information on the questions included in each index and the outcome variables can be found in Tables 1 and 2. Scoring information can be found in the supplementary information section along with score distributions for each constraint type.

**Table 1:** Demographics and menstruation-related constraints on educational outcomes.

Variable	Mean	Std. Dev.	Min.	Max.	N
***Panel A: Demographics***					
Age	15.299	1.434	13	22	441
Grade	1.715	0.715	1	3	502
Duration of residence	7.359	5.879	0.1	24	448
Age started school	6.818	2.051	2	18	479
Have you had your period? (Yes = 1)	0.862	-	0	1	515
Age at first period	14.155	1.131	9	19	444
***Panel B: Absenteeism and Participation***					
*This panel samples the 86% of girls who have had their period*					
On last period, missed school bc of period (Yes = 1)	0.326	-	0	1	445
Number of days missed during last period	2.656	1.492	1	7	131
On last period, left school early bc of period (Yes = 1)	0.467	-	0	1	418
On last period, did not participate as much as normal (Yes = 1)	0.311	-	0	1	425
On last period, did not concentrate as much as normal (Yes = 1)	0.33	-	0	1	430
***Panel C: Menstrual Knowledge***					
How often does a girl get her period? (1 = correct)	0.507	-	0	1	523
How long does a period last, on average? (1 = correct)	0.725	-	0	1	523
At what age do girls generally get their period? (1 = correct)	0.786	-	0	1	523
At what age do women stop getting their period? (1 = correct)	0.692	-	0	1	523
Can a girl get pregnant after reaching menarche? (1 = correct)	0.922	-	0	1	523
Is menstrual blood the shedding of the endometrium lining? (1 = correct)	0.631	-	0	1	523
Does a menstrual period indicate a woman is not pregnant? (1 = correct)	0.719	-	0	1	523
Does ovulation occur 14 days before ones’ period? (1 = correct)	0.484	-	0	1	523
Does ovulation occur during one’s period? (1 = correct)	0.294	-	0	1	523
Is physical discomfort common during menstruation? (1 = correct)	0.837	-	0	1	523
Menstrual knowledge score index	0.66	0.181	0	1	523

Notes: This table provides information on sample demographics, rates of absenteeism and participation. Standard deviations presented for continuous variables only. Sample sizes vary in **Panel A**, since students were not forced to respond. **Panel B** is for a base sample of 444 students who reported ever having had their period. Reduced sample size from 444 is due to attrition, with the exception of the second row limited to those that missed at least one day of school during their last period. **Panel C** includes a summary of the questions that were included in the menstrual knowledge quiz portion of the survey. The mean value indicates the percentage of girls that correctly answered each question. The final index value shows girls’ final knowledge scores when all questions are taken into consideration.

**Table 2:** Demographics and menstruation-related constraint indices.

Variable	Mean	Std. Dev.	Min.	Max.	N
**Panel A. Information on menstruation: sources and content**					
Received info: health worker (Yes = 1)	0.797	-	0	1	395
Received info: school curricula (Yes = 1)	0.759	-	0	1	349
No. of people who gave info on menstruation	4.099	3.027	0	16	523
Discussed periods with (#) of 5 closest female friends	2.116	1.766	0	5	466
*If I received advice, I received advice on…*					
how to use towels/fabrics/linen (Yes = 1)	0.899	-	0	1	404
how to stay clean/wash (Yes = 1)	0.833	-	0	1	348
how to manage pain (Yes = 1)	0.605	-	0	1	276
things/activities to avoid during menstr. (Yes = 1)	0.772	-	0	1	325
***Informational Constraint Index Questions:***					
Someone told me about periods and why they happen (Yes = 1)	0.902	-	0	1	447
Someone told me how to manage menstruation (Yes = 1)	0.951	-	0	1	509
**Panel B1. Socio-Cultural: shame and restrictions**					
*This panel samples the 86% of girls who have had their period*					
Would feel shame if other girls knew about my period (Yes = 1)	0.37	-	0	1	497
Would feel shame if boys knew about my period (Yes = 1)	0.844	-	0	1	493
Menst’ing women restricted in respondent’s home (Yes = 1)	0.416	-	0	1	442
**Panel B2. Socio-Cultural: fear**					
Fear teachers not understanding/helpful (Yes = 1)	0.258	-	0	1	489
***Socio-cultural Constraint Index Questions:***					
*If people knew that I am post-menarche, I would fear…* (Yes = 1)					
…teasing	0.576	-	0	1	490
…touching against will or harassment to go out with someone	0.696	-	0	1	470
…unwanted pregnancies or dishonor	0.868	-	0	1	500
…pressure to marry/take a boyfriend	0.641	-	0	1	476
**Panel C. Biological: preparedness and pain**					
*This panel samples the 86% of girls who have had their period*					
Period duration (days)	3.781	1.255	1	14	433
Respondent keeps track of menst. cycle (Yes = 1)	0.732	-	0	1	429
Time between bleedings varies a lot (Yes = 1)	0.62	-	0	1	432
Hard to know/prepare for bleeding (Yes = 1)	0.602	-	0	1	442
Physical pain rel. to friends	2.694	1.755	1	5	448
Always/sometimes use painkillers on per. (Yes = 1)	0.485	-	0	1	406
Bleeding rel. to friends	2.894	1.481	1	5	443
***Biological Constraint Index Questions:***					
Degree bleeding (1–5)	2.562	1.653	1	5	441
Degree physical pain (1–5)	3.101	1.788	1	5	447
**Panel D. Material: affordability and accessibility**					
Use cloth most often (Yes = 1)	0.466	-	0	1	479
Use natural materials most often (Yes = 1)	0.426	-	0	1	479
Use pads most often (Yes = 1)	0.071	-	0	1	479
Use nothing most often (Yes = 1)	0.025	-	0	1	479
Prefers pads (Yes = 1)	0.215	-	0	1	489
Prefers natural materials (Yes = 1)	0.538	-	0	1	487
Monthly expenditure (1000 THS)	6.677	17.361	0	300	360
Monthly expenditure on pads (1000 THS)	4.541	5.743	0	70	377
Can wash body at home (Yes = 1)	0.962	-	0	1	496
***Material Constraint Index Questions:***					
Always uses preferred method (Yes = 1)	0.735	-	0	1	471
Do you feel safe in the latrines at school during your period? (Yes = 1)	0.174	-	0	1	482

Notes: This table provides summary statistics about information, biological, material, and socio-cultural constraints as experienced in the sample. In addition, the table provides insight into the questions used to develop each constraint index–please see the bold and italicized headings within each panel.

For each educational outcome (dependent variables) the regression controls for all four constraint types (independent variables). Therefore, we are able to measure the relative impact of each constraint on girls’ educational outcomes.

$$\begin{array}{c} Y_{s}=\beta_{0}+\beta_{1}SocioCulturalConstraint_{s}+\beta_{2}BiologicalConstraint_{s}\\ +\beta_{3}MaterialConstraints_{s}+\alpha_{is}+\varepsilon_{s} \end{array}$$ (1)

In the equation above, Y represents each of the four educational outcomes (missing school, leaving early, concentration, and participation). Every constraint index is represented in the equation which allows us to take into account each constraint type’s relative impact on the outcomes of interest. The equation includes school fixed effects, *s*, and controls for a vector of individual level characteristics, *α*_*i*_ (age, grade, and age at menarche).

In summary, the socio-cultural constraint index captures the prevalence of girls’ common menstruation-related fears, the biological constraint index captures experiences with physical pain and bleeding due to menstruation, and the material constraint index captures access to and experiences with proper MHM.

Although informational constraints are discussed throughout the paper, they are not included in the final model due to a lack of reasonable variation. We find that girls in the sample are generally well-informed about menstruation and that it is rare for them to experience any significant informational constraints. Therefore, the index is omitted to prevent the introduction of bias into the model and the other results. Moreover, the number of questions included in each of the remaining indices was limited by sample size considerations, including too many questions with differing rates of attrition caused sample sizes to fall drastically. Therefore, we only include the most relevant questions for each constraint type in the index. Distributions for each constraint index, including informational, can be found in S1 Fig. For transparency, the original regression that includes the first iteration of the material constraint index, the informational constraints index and the knowledge score are available in the supplementary information section, as well as S2 and S3 Figs.

## Results

**Fig 1:**
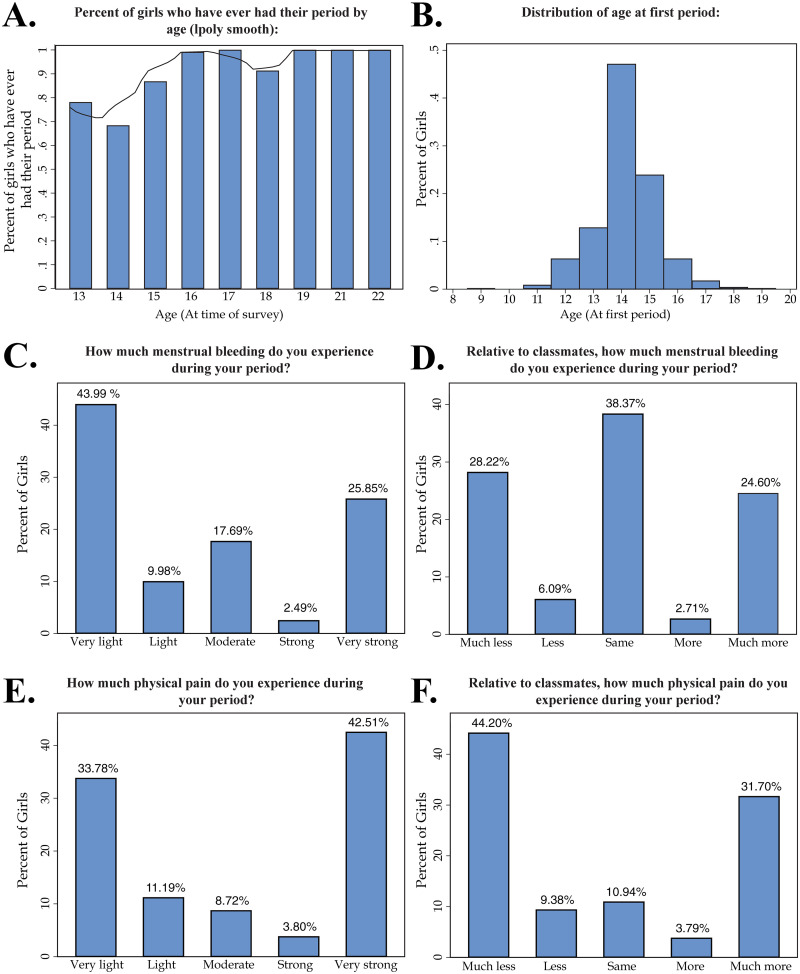
Age of menarche and degree of pain and bleeding during menstruation. The sub-figures included provide relevant information regarding the samples’ experiences with pain and bleeding during menstruation as well as age of menarche. **A)** The percent of girls who have ever had their period separated by their age during surveying. The figure includes a local polynomial smooth. **B)** A histogram showing the distribution of age at first period. **C)** “How much menstrual bleeding do you experience during your period?” **D)** “Relative to classmates, how much menstrual bleeding do you experience during your period?” **E)** “How much physical pain do you experience during your period?” **F)** “Relative to classmates, how much physical pain do you experience during your period?”.

Eighty-six percent (86%) of female respondents were post-menarche at the time of survey and the average age of menarche, using the recall method, was 14.2 years-old (Table 1, Fig 1). Though data on age of menarche in Tanzania is sparse, this estimate closely matches the average age found in a 2005 (the most recent study identified by the authors) study of 71 girls in the Dar es Salaam and Mafinga regions of Tanzania [57]. Further, on average, girls in the sample were 15.2 years-old, started school at 6.8 years-old, and had resided in their current residence for 7.4 years (Table 1).

Table 1 reports summary statistics on the four educational outcomes of interest––absenteeism, leaving school early, participating less, and concentrating less. One-third (33%) of respondents reported that they missed at least one school day during their last period because of their period. Among this group, the average number of school days missed was 2.7 (Table 1). Notably, a significantly larger proportion of girls (47%) reported that they left school early during their last period because of their period. We also find that girls’ self-reported academic performance was affected by menstruation. Thirty-one percent (31%) report that they could not participate as normal and 33% could not concentrate as normal during their last period (Table 1).

### Informational constraints

Contrary to much prior qualitative research in developing countries [7, 28, 32, 58], we find that overall, girls in the sample received detailed, high-quality information from multiple sources about the biological basis and management of menstruation. A clear majority of girls reported that someone had told them about periods and why they happen (90%) and that someone had taught them how to manage menstruation (95%) (Table 2, Panel A). Further, girls received information from an average of 4.5 sources, ranging from a list of relatives and friends to school, health, and religious officials (Table 2). We verify that the information was high quality, as 80% received information from health workers, and 76% from school curricula. This mitigates concerns that information from family and friends can propagate misconceptions or stigmas [59] (Table 2, Panel A). We find that girls also received practical information as more than 80% reported they’d received advice on how to use menstrual absorbents and how to wash themselves and stay clean (Table 2, Panel A). Thus, the cohort had a high level of informational preparedness.

While the overall results on informational constraints are optimistic, we find evidence that some girls slip through the cracks, and that proscriptions (restrictions on menstruating women) remain. Ten percent of girls in the sample (10%) had not been told about the causes of periods, yet further investigation revealed that 86% of the group were post-menarche. Therefore, it is possible that the girls who had yet to be informed about menstruation were not also awaiting their first period. Girls in this “uninformed” group were less likely to report receiving information on menstrual management (86%) or information from health workers (63%) and school curricula (54%). As a result, there exists a cohort of girls who have received below-average levels of information across many different measures. This indicates that there is still room for improvement in educating young women about menstruation, even in settings that have a fairly high level of informational preparedness.

Second, although girls received valuable advice on menstrual management, 77% also reported that they had been advised to avoid certain objects or activities during menstruation (Table 2, Panel A). In summary, while girls generally receive detailed practical information regarding absorbents and washing, the information is commonly accompanied by ideas that, whether harmfully or not, imply that menstruating girls should deviate from normal behavior. This practice can perpetuate stigmas and lead to socio-cultural anxieties related to menstruation. Consequently, it is essential that the impact of menstruation-related socio-cultural constraints on girls’ well-being and education is explored further.

### Socio-cultural constraints

**Fig 2:**
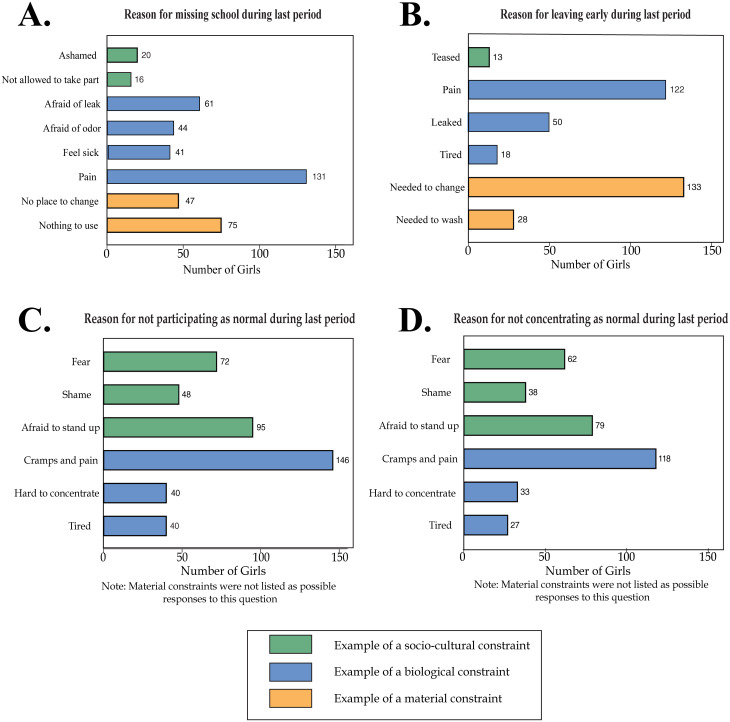
Reasons for absenteeism and reduced participation. The sub-figures indicate the prevalence of each reason, as selected by girls in the sample, for absenteeism or reduced educational outcomes. Please note, the reasons listed here, although coded by constraint type, are not the questions used to build the corresponding constraint index. **A)** Reason for missing school during last period; **B)** Reason for leaving early during last period; **C)** Reason for not participating as normal during last period; **D)** Reason for not concentrating as normal during last period. Please note the underlying data in this figure was previously published in Benshaul-Tolonen et al. (2020) [39].

*Socio-cultural* constraints related to menstruation inhibit girls’ educational outcomes. Apart from cramps and pain, shame and fear are the two most prevalent reasons for reduced participation and concentration while in school. Roughly 134 girls in the sample cite fear and 86 girls cite shame as the reason for their change in participation or concentration (Fig 2). The question naturally follows: If shame and fear play such a large part in girls’ desires to hide their period in school, then what is the source of this shame?

Through the survey, we investigate different types of teasing and harassment that have previously been linked to menstruation in qualitative research, but have rarely been quantified. We identify and study three overlapping types of negative social consequences for revealing one’s period: sexualization, a gendered sense of impropriety, and stigmas outside of peer interactions.

First, the vast majority of girls fear harassment borne from attitudes that associate menarche with physical and/or sexual maturity and marriageability. We asked girls about their fears regarding specific forms of harassment, if people knew they were post-menarche. Seventy percent (70%) of girls in the sample fear being touched against their will or harassed to go out with someone, while 87% fear unwanted pregnancies or dishonor and 64% fear the pressure to marry or take a boyfriend (Table 2, Panel B). These questions are used to develop our socio-cultural constraint index as they capture different forms of fear and shame that girls may associate with menstruation.

Second, the sample demonstrates a gendered understanding of appropriateness in revealing one’s period, both at school and in the home. While a substantial portion of girls (37%) report that they would feel shame if girls at school knew about their periods, a much larger portion (84%) would feel shame if male peers knew (Table 2, Panel B). We conjecture that menstruation-related shame is partly derived from this gendered sense of appropriate confidants for female-specific issues, and partly due to the fear of possible public shaming. Furthermore, 58% of girls fear being teased if people knew they were post-menarche. This supports our claim that fear of public shaming and perceived appropriateness are major factors in menstruation-related shame. In addition, we have previously reported that male peers are the most commonly feared perpetrators of period teasing among this sample of girls [39].

Third, girls are subject to socio-cultural constraints related to menstruation in their interactions with teachers and family members. For instance, twenty-six (26%) of girls fear teachers being unhelpful or not understanding if they knew a student was post-menarche. Likewise, 38% of girls report that menstruating women are restricted from certain daily activities in their homes (Table 2).

In conclusion, girls in the sample express both shame of revealing menstrual blood or their period status, and fear of harassment related to the sexualization of post-menarche girls. Given evidence that period-related teasing is common in the sampled schools, we conjecture that girls’ fear and shame are consequences of witnessing post-menarche girls suffer negative social interactions. Family, teachers, and friends may also instill fear by transmitting social norms, attitudes, and beliefs about real or imagined consequences of revealing menstruation, though our survey was not designed to trace the origin of these ideas. Accordingly, these aspects of shame and fear then predominantly contribute to girls’ absenteeism, decreased participation, and decreased concentration in school during menstruation. Thus, fear and shame are plausibly consequences of socio-cultural constraints on menstruating women, but in turn they also cause girls to constrain their own social behavior.

### Biological constraints

In general, girls report that they have irregular periods which they find difficult to manage, signaling both biological and material constraints. In terms of biological constraints, 73% of girls reported that they keep track of their menstrual cycle, however, 62% of girls report that the time between their periods varies heavily (Table 2, Panel C). This indicates that even though girls may be familiar with cycle tracking techniques, the irregularity of menstruation can pose a threat to their ability to prepare for their period. Accordingly, 60% of girls, in the sample, reported that it is hard to anticipate and/or prepare for their next period (Table 2, Panel C). The sample reported an average degree of bleeding during menstruation of 2.6 (1 being no bleeding, 5 being very strong bleeding) (Table 2, Panel C and Fig 1). Additionally, for some, cramps (dysmenorrhea) and pain are prominent physical symptoms during menstruation.

When asked to judge their level of physical pain during menstruation (1 being no pain, 5 being very strong pain), the girls reported an average pain level of 3.1 (Table 2, Panel C and Fig 1). Correspondingly, “cramps and pain” is cited as the most or second-most common reason for all four educational outcomes (Fig 2). For instance, of those girls that missed a day of school during their last period, over 100 reported that they missed school because of “cramps and pain”—by far the most prevalent reason. Similar results are seen across educational outcomes––cramps and pain are the most or second most prevalent reason for leaving early, participating less, and concentrating less (Fig 2). Therefore, biological constraints related to menstruation can act as a serious impediment to girls’ educational outcomes. Furthermore, we find that 49% of girls in the sample always or sometimes use painkillers during menstruation (Table 2, Panel C). This finding emphasizes how debilitating symptoms of premenstrual syndrome can be to young women and that some may be reliant on pain medication to manage their period symptoms.

As a result, the questions regarding degree of physical pain and bleeding were used to develop the biological constraints index. The index aims to capture the degree at which each girl is affected by these biological constraints to determine their relative impact on each educational outcome.

### Material constraints

**Fig 3:**
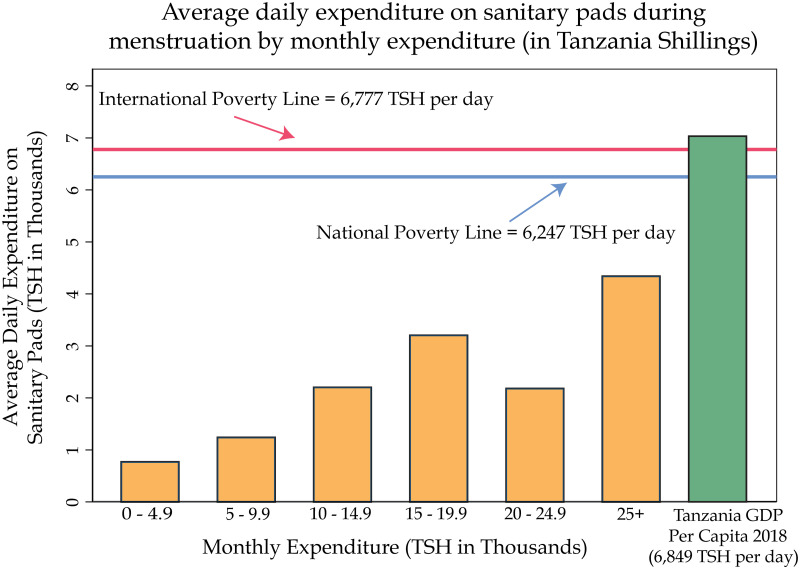
Monthly expenditure and spending on sanitary pads. This figure presents girls’ average daily expenditure on sanitary pads during menstruation (self-reported monthly expenditure on sanitary pads expressed in 1000 TSH, divided by the average length of menstrual period) by their monthly self-reported expenditure (ranked and expressed in 1000s TSH). In addition, the *green bar* shows the approximate daily value of Tanzania’s 2018 GDP per capita [60] (approximately equivalent to 2.9 USD per capita), the *blue line* presents Tanzania’s national poverty line [61], the *red line* indicates the international poverty line [62].

Although prior research suggests that material constraints may largely affect young women’s ability to obtain their preferred MHM absorbents, the majority (73%) of girls in our sample report that they always use their most preferred method. As noted in Table 2, Panel D, 47% of girls most often use cloths as menstrual absorbents, though only 23% prefer cloth. Likewise, the majority of girls (54%) would prefer to use natural materials (which may include raw cotton but also grass, leaves, and cow dung), but only 43% use natural materials most often. In the survey, girls were not asked to specify what they meant as “natural material”, therefore, we are unable to provide a breakdown of this category. However, the largest disparity between use and preference is in relation to pads: 21% of girls would prefer to use pads, but only 7% use pads most often. We theorize that pad affordability is the primary cause of this deficit. Of the girls who did not use their most preferred method during their last period, 60% cited “not enough money” as one reason. Further, it is notable that the average girl spends more than two-thirds of her total monthly expenditure on pads (Table 2, Panel D). Fig 3 depicts average daily expenditure on sanitary pads during menstruation by girls’ monthly expenditure and compares spending to the national poverty line, international poverty line, and GDP per capita. The results show that average daily expenditure on sanitary pads is higher as girls’ monthly expenditure increases. Daily expenditure on pads was calculated using girls’ stated monthly expenditure on pads and the average menstrual cycle length in the sample (3.8 days). Through these calculations, we find that girls in the sample are spending anywhere from 12–70% of the national poverty line (6,247 TSH per day) on pads during their period (Fig 3). The second most prominent material constraint, with respect to using one’s preferred absorbent, is lack of availability. Over 25% of girls who did not use their most preferred method last month cited “method unavailable” as a reason (Table 2, Panel D). As a result, we developed the material constraint index using the question regarding using one’s preferred method and a separate question on one’s ability to wash their body at home.

Aside from cramps and pain, material constraints such as accessing absorbents and school latrine infrastructure are the main reason why girls miss school or leave school early according to their survey responses (Fig 2). In the survey, we asked girls to choose every applicable reason for why they have missed school, left early, or experienced changes in participation and concentration. Prevalent reasons for missing school included “nothing to use” (75 girls) and “no place to change” (47 girls). Similarly, aside from cramps and pain, the most common reasons for leaving school early are “needed to change” (133 girls) and “needed to wash” (28 girls) (Fig 2). These results, indicate that a variety of material constraints prevent girls from attending school including insufficient access to absorbents or reliable WASH facilities. Correspondingly, the responses confirm a lack of sufficient infrastructure at school for changing and washing.

Furthermore, girls’ ability to afford or acquire pain medication may interact with their ability to manage menstrual pain which is commonly cited as a reason for menstruation-related absenteeism. Other biological constraints such as fatigue or sickness were less commonly reported. Put simply, access to pain medication, a material constraint, may impact girls’ educational outcomes. However, capturing the interaction between material constraints and biological constraints is beyond the scope of this paper and the relationship should be explored in future research.

### Correlates of absenteeism and other impaired educational outcomes

**Fig 4:**
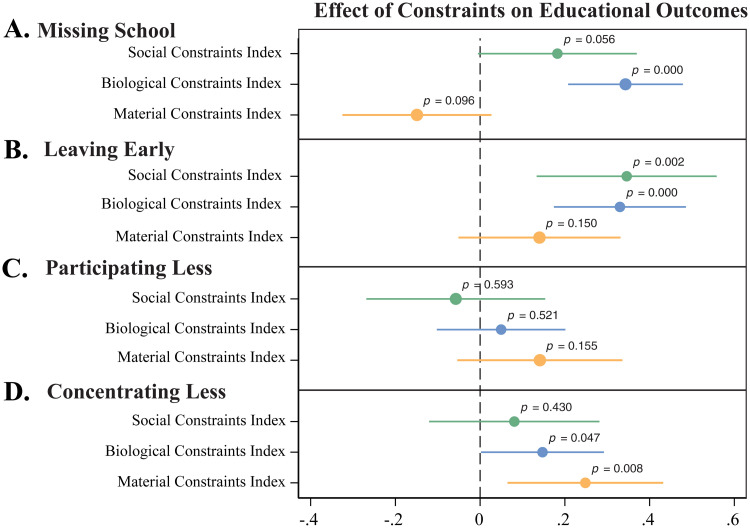
Regression results: Effect of constraints on educational outcomes. The figure above depicts plotted regression coefficients for each constraint type and its relationship with each educational outcome of interest. The model includes three constraint types: social, biological, and material and four educational outcomes: **A)** “During your last period, were there days when you could not come to school because of your period?” **B)** “During your last period, did you ever leave school early because of your period?” **C)** “During your last period, did you participate as much in class as you normally do (when you do not have your period)?” and **D)** “During your last period, did you concentrate as much in class as you normally do (when you do not have your period)?”. In addition, the figure includes p-value labels for each coefficient to indicate significance.

In Fig 4, we investigate whether material, social, and biological constraints are significantly correlated with absenteeism, leaving early, decreased participation, and decreased concentration. The three categories of correlates tested—material, social, and biological—were chosen based on the four theories of constraints previously discussed. Ultimately, we form three conclusions of interest. First, under the logic of material constraints, girls who are unable to access or afford their preferred absorbent and/or are unable to wash their body at home suffer from inadequate MHM which may negatively impact educational outcomes. Second, according to the logic of socio-cultural constraints, girls who experience more menstruation-related shame or fear may be less comfortable in social settings during menstruation, and thus have more significant educational impediments. Finally, in the logic of biological constraints, girls who experience more physical pain or a greater degree of bleeding during menstruation may be worse equipped to manage or conceal their physical symptoms resulting in considerable obstacles to education.

Comprehensive descriptions of each index and scoring guidelines can be found in the previous section, in Table 2, and in the supplementary information section.

We find substantial support for the socio-cultural constraints theory in relation to absenteeism and leaving school early. Girls who experience more socio-cultural constraints are significantly more likely to stay home or leave school early during their period and because of their period, (*p* < 0.10) and (*p* < 0.01) respectively. For example, in the fully identified model, girls who reported a socio-cultural constraint index score of 1 were 18.2 percentage points (56% of the sample mean) more likely to miss school (Fig 4) and 34.6 percentage points (74% of the sample mean) more likely to leave school early (Fig 4). However, the regression coefficient between the social constraint index and participating or concentrating in class is not statistically significant. Therefore, we conclude that shame, fear and the socio-cultural constraints that drive them, contribute to absenteeism more than the other educational outcomes.

Likewise, there is a significant association between biological constraints and our educational outcomes of interest. Girls who are more biologically constrained, those who experience more pain or bleeding during menstruation, are significantly more likely to stay home, leave school early, and concentrate less in class, (*p* < 0.01), (*p* < 0.01), and (*p* < 0.05) respectively. In the fully identified model, girls who report a biological constraint index score of 1 were 34.3% more likely to miss school (105.2% of the sample mean), 33% more likely to leave early (70.7% of the sample mean), and 14.7% more likely to concentrate less in class (44.5% of the sample mean) (Fig 4). These results indicate that biological constraints have a considerable impact on girls’ educational outcomes, especially on absenteeism.

Finally, the results demonstrate that material constraints play a similar key role in determining educational outcomes. In the sample, girls who experience more material constraints are significantly less likely to miss school and are more likely to concentrate less in class, (*p* < 0.10) and (*p* < 0.01) respectively. In the fully identified model, girls who report a material constraint index score of 1 were 14.9% less likely to miss school (45.7% of the sample mean) and 24.8% more likely to concentrate less in class (75.2% of the sample mean). These results indicate that material constraints may have a greater impact on educational outcomes like participating and concentrating in class than previously anticipated. Furthermore, it emphasizes the importance of access to MHM absorbents and WASH facilities.

## Discussion

### Main results

Through this exercise, we found abundant evidence that menstrual hygiene management impacts educational outcomes. One third of girls stated that they missed at least one day of school during their last period, while nearly half of the girls voiced that they left school early because of their period. Moreover, we report significant rates of decreased participation and concentration in the classroom as menstruation-related educational impediments. Although absenteeism may represent more serious educational losses, our results indicate that limiting research to these outcomes can overlook significant barriers to educational access among adolescent girls. We investigate the causes of these educational impediments by breaking up potential constraints into four categories: informational, socio-cultural, biological, and material.

Three principal conclusions are derived from this study: i) interventions focused on the provision of menstrual absorbents should consider including participation and concentration as primary educational outcomes, ii) material constraint interventions should expand their focus to adequate WASH infrastructure, as it significantly impacts early departure, and iii) to date, socio-cultural constraints (in particular, fear and shame) and biological constraints (in particular, dysmenhorrea) are seriously understudied in quantitative research although they emerge as primary drivers of girls’ absenteeism from school.

These results present a relevant addition to the insights generated by previous correlational findings in the literature. For instance, the study conducted in Uttar Pradesh by Malhotra et al. (2016) [26] finds that two-thirds of the girls reported constraints in the management of menstruation. This figure represents a benchmark for this study, which revealed that 77% of girls had been restricted from doing certain activities during menstruation. Nevertheless, our study differs in many aspects, including regional focus and a different approach to household factors. While we focus on direct factors of MHM access, such as the ability to afford the products and washing infrastructure at home, their study looks at indirect correlations of MHM, including household economic and demographic characteristics. Overall, more research that encompasses all aspects of material constraints is needed to determine their relative importance.

#### Informational constraints

The surveyed adolescent girls report receiving high-quality information about the biological basis and management of menstruation from multiple sources ranging from family to school curricula. This is in contrast to earlier evidence that girls in developing countries, including the East African region [5] and Tanzania [4], are not adequately informed about menstruation or basic MHM techniques. With the caveat that our school sample is a convenience sample and not representative of the region, this could highlight recent improvements in MHM curricula and adolescent girls’ access to alternative sources. The external validity of this result should be investigated in more comprehensive and representative samples.

We do, however, find a small minority of girls, fewer than one in 10, who have slipped through the cracks. These girls are less likely to have received information on MHM prior to menarche and are under-exposed to what we deem are high-quality sources of information. Reaching this population which has reduced exposure to information from sources across the board may demand targeted interventions, similar to reaching out-of-school adolescent youth.

#### Socio-cultural constraints

We argue that socio-cultural constraints—in particular, stigmas surrounding menstruation that generate shame and fear in girls—are a prominent barrier that prevents students from attending, participating, and concentrating in class. We offer the first quantitative evidence to confirm previous qualitative findings in the region [4, 7, 39] that teasing and harassment are widely feared among girls. Often, menstruation-related teasing and harassment can be linked to the sexualization of young women as some perpetrators associate the onset of puberty with physical maturity. Girls fear non-consensual touching and pressure to date (70%), unwanted pregnancies or dishonor (87%), and pressure to marry or take a boyfriend (64%). Further, girls generally fear revealing that they are on their periods to male classmates (84.4%), but some even fear revealing it to their female peers (37%). This result aligns with the findings in Benshaul-Tolonen et al. (2020) [39] that boys, in the same schools, generally believe it is inappropriate for girls to discuss periods with male classmates, male teachers, and fathers, alluding to a symmetrical stigma hindering discussions of these topics across genders. Girls may fear that boys will engage in period teasing, which is often prompted by odors and leaks that publicly reveal a girls’ menstruation [1, 39, 63, 64]. Correspondingly, 58% of girls in the sample fear teasing if their menstrual status is revealed. Our results confirm previous qualitative research [5] by finding that 38% of girls report being restricted from certain daily activities in their home while menstruating. As a result, proscriptions may carry-over negative attitudes toward menstruation from the household into the classroom and lead to teasing [39] or shame [7].

Lastly, we are able to quantify the effect of our proposed operationalization of socio-cultural constraints on several educational outcomes including absenteeism, decreased participation, and decreased concentration. Increased perception of socio-cultural constraints is significantly associated with increased risk of missing school and leaving early––two serious impediments to young women’s educations. Socio-cultural interventions should target the broader community, including male students, families, and educators, to be most effective [7, 23, 32, 39, 65]. Previous research has explored using drama skits [23] and games [65] to combat social stigmas associated with puberty and menstruation within communities. Combined with the aforementioned results, we are able to conclude that socio-cultural constraints, although they are often overlooked, ought to be included in MHM interventions.

#### Biological constraints

This study contributes rare quantitative findings to a nascent body of evidence that menstrual cramps and pain are major causes of girls’ absenteeism [8, 13, 15, 19, 29, 30, 66] and their impaired participation/concentration in school activities [31]. Among our sample of 524 girls, cramps and pain are consistently the most or second-most prevalent reason for all four adverse educational outcomes: absenteeism, leaving early, and reduced participation and concentration. Roughly one third of girls listed cramps and pain as a determinant, for all four outcomes. This is in line with the descriptive statistics showing a largely bimodal distribution in dysmenhorrea: girls report either experiencing very light pain, or very strong pain, rather than moderate pain. In parallel, they report experiencing much less or more pain that their classmates, rather than the same. Because dysmenhorrea is not universal, a pain alleviating intervention may have strongly heterogeneous effects, but provide important respite from cramps and pain for a subset of the sample.

Combined, these results enforce previous calls to consider pain management techniques [15, 29, 67], including, but not limited to, the provision of painkillers as a potentially important element in MHM interventions. Evidence from the Meniscus-2 intervention [23] reiterates the importance of educating secondary school students on pain management techniques and providing alternatives to painkillers. Each girl in the Meniscus-2 study received a voucher for 6 paracetamol tablets per month: 25% of girls redeemed the vouchers, which could signal low preference for analgesics, or alternatively, uptake only among a subset of girls who experience strong cramps and pain.

Regularly acquiring and utilizing painkillers can pose a financial burden on menstruating women and their families, which, acts as an additional material constraint. Moreover, cultural practices may affect girls’ willingness to utilize analgesics. Simply put, girls with less access to material resources or those with strict cultural values against pain-medications will be further disadvantaged in their ability to manage menstrual pain leading to deeper inequalities. As a result, the relationship between biological constraints, pain management, and material constraints cannot be ignored. We propose future policy evaluations to include vouchers for painkillers as well as alternative pain management techniques.

The girls report not being equipped to understand their own menstrual cycles. Over 60% of girls voice that it is difficult to know when their periods will occur, making it challenging to prepare for menstruation. Likewise, 62% of the sample state that time between each cycle can vary substantially. These findings directly align with previous research that irregular menstruation is likely to occur during early adolescence, especially the first 1–2 years after menarche [68]. Girls who are unable to prepare for menstruation may experience more problems with leaking, odors, and general MHM (material and biological constraints) than their peers with regular menstrual cycles. Furthermore, it has been found that menstrual irregularities are correlated with social status and location––girls in rural areas are more likely to experience irregular periods [69].

Women in high-income countries commonly engage in cycle tracking to understand and prepare for their menstrual cycle, spot any irregularities, and prepare for or avoid conception [70, 71]. The reasons to use cycle tracking may also apply to adolescents in Tanzania. Indeed, 73% of girls in the sample try to monitor their menstrual cycle, but their preferred tracking methods are unknown (e.g. pen and paper, technology, etc.), and despite this, they report issues with predicting their cycle month-to-month. Previous research has analyzed the efficacy and accuracy of different methods of cycle tracking, ranging from using technology to memory [70, 72–74]. Future interventions should provide sophisticated menstrual tracking resources to adolescent girls to understand their implications for educational outcomes, taking into account equity concerns about smartphone health applications [75].

#### Material constraints

Third, we uncover some disparity in girls’ desired absorbents compared to their accessed absorbents. A significant portion of girls use cloth as a next-best option, since 47% of girls use cloth most often but only 23% prefer to do so. We argue that girls are unable to use their most-preferred absorbent due to accessibility and affordability barriers. Somewhat contrary to the dominant narrative in MHM advocacy for providing girls with pads (reusable or disposable), menstrual cups, and tampons, 54% of girls in our sample prefer to use natural materials. This result may reflect the general availability of natural materials, as nearly 43% of girls are able to use them most often. However, since many girls report anxiety around disposing menstrual absorbents and revealing their menstruation [38], this result may reflect the easy and discreet nature of disposing natural materials. For those who prefer to use pads, affordability appears to be the binding constraint. Our results show that only 7% of girls use pads most often, but that 21% of girls would prefer to use them.

Inaccessibility of safe leak-proof absorbents and inadequate WASH facilities are common material constraints that cause girls to stay at home or leave school early. A large portion of the sample, over 100 girls, reported that they had stayed home during their last period because they had nothing to use or they had no place to change. Likewise, over 150 girls, reported leaving school early during their last period because they needed to wash or change. We find considerable evidence that material constraints significantly increase the likelihood of leaving school early, impairing participation, and decreasing concentration. The results are similar to findings from other developing countries (e.g. [30] in rural Ghana; [15] 2018 in peri-urban Uganda; [31] in India) that nonavailability of absorbents and the lack of private places to manage periods at school are major reasons for menstruation-related absenteeism. On average, girls, in the sample, spend two thirds of their total monthly expenditure on pads. Further, we find that daily spending on sanitary pads during menstruation is higher with increased monthly expenditure: poorer girls have limited opportunity to access commercial sanitary products. However, the girls spend between 12–70% of the national poverty line (6,247 THS daily) on sanitary pads, signaling undue financial burden on adolescent girls and indicating that the provision of free sanitary products will have significant financial impacts on girls’ and households’ budgets. Affordability constraints are particularly interesting given that boys in the same schools have much higher monthly expenditure (on average 3.2 times higher) than girls [39], and the difference in expenditure is statistically significant (*p* < 0.01).

Our results are in line with findings from menstrual absorbent interventions –– material constraints, as measured by the material constraint index (use of preferred absorbent and school latrine safety) do not have a clear negative impact on girls’ absenteeism [18–20, 76]. Our evidence instead suggests that material constraints impact girls’ concentration in the classroom. This aligns with recent evidence from a randomized control trial in Kenya that providing girls with sanitary pads or menstrual cups improves well-being but not attendance [20]. However, our findings are limited in their interpretation as they only directly relate to the questions utilized in the material constraint index.

The expansion and development of WASH facilities could significantly improve girls’ educational outcomes. Girls in the study commonly reported “needed to wash” and “needed to change” as reasons for leaving school early. This finding signals that inadequate access to WASH facilities may pose a more significant material constraint in terms of early departure. Although limited, previous research focused on the development of WASH infrastructure has shown promising results [24, 55, 77] which support our findings.

In order to combat the impact of material constraints on educational outcomes, we propose further research on the improvement of WASH facilities and girls’ preferences regarding menstrual absorbents. Moreover, future studies should include a longer list of questions than utilized in this survey to more broadly capture girls’ experiences with material constraints.

### Study weaknesses

The methodology of this study has several weaknesses. First, the results presented here cannot be interpreted as representative of Tanzania or the Geita and Sengerema Districts, because the four public secondary schools were selected for convenience. For the same reason, we were unable to intentionally target multiple types of schools, e.g. private, public, boarding or day schools. However, three schools were day schools and the fourth was a boarding school. All schools in the sample were mixed-gender.

Second, cost prohibited including a sample larger than roughly 500 girls, and the sample size was not calculated *a priori* to achieve specific statistical power. The exercise was intended as a pilot study to prepare for a larger randomized control trial, and is thereby limited in scope.

Third, our sample is restricted to girls currently enrolled in and attending school. As many qualitative studies have highlighted (e.g. [12, 14, 48, 78]), girls who have dropped out of school have different experiences with menstruation and are less likely to learn about it from school curricula. While school participation and absenteeism are our main outcomes of interest, we do not capture the more extreme consequence of school drop-out. Moreover, our study finds that age of menarche occurred slightly earlier in our sample relative to previous research. Therefore, we urge future studies or interventions to include younger girls to accurately capture the impact of access to menstrual hygiene on school dropout.

Fourth, we did not include the informational constraint index in the final linear analysis model. The constraint was omitted due to a lack of real variation in the sample. If it were included, the index would have introduced bias to the model and the remaining regression results. As a result, we are unable to consider how informational constraints impact girls’ educational outcomes in conjunction with the other constraint indices. However, alternative measures of information access, which we did not collect, could possibly yield meaningful variation in the data. Therefore, we encourage future research to consider applying this methodology to samples that naturally experience more informational constraints and expand on this finding. Nevertheless, we ran the analysis using two separate indexes for knowledge (previously defined in [39]) and information and present the results in S2 Fig. We find that the score on the knowledge index does not have a statistically significant correlation with our four educational outcomes.

Fifth, the regression results are limited in their interpretation due to the small sample size and inability to include extensive control variables due to survey formulation. In addition, the number of observations is affected because many of the girls in the sample have missing values for at least one of the question used in the indices. Therefore, it is possible that our regression outcomes are not fully representative of the study population. It may be that girls who perceive a particular question as socially sensitive are less likely to respond, thereby skewing the results. We cannot test for this but encourage future studies to bear this in mind when designing the questionnaire.

Sixth, as described in the background section, the domains of constraints on MHM –informational, socio-cultural, biological and material– were constructed following a review of the past literature. They map onto multiple existing frameworks for understanding menstrual health, are broadly comparable to UNICEF’s pillars of menstrual health interventions (2019) [11], and reflect many of the themes in the integrated model of menstrual experience [22] and the domains proposed by Plesons et al. (2021) [10]. However, the questions encompassed in each constraint were not chosen a priori to the data collection, but were included post-hoc. The post-hoc development of the indices, in some instances, constrains our ability to interpret the results, as the questions were not developed for this purpose. Post-hoc selection of questions can also allow researcher cherry picking of statistically significant results [79], an issue that we are aware but tried to avoid. For transparency, (1) we only selected questions that are clearly linked to each constraint type; (2) Table 2 shows the questions that were not selected due to the researchers’ priors; and (3) the exclusion of questions from the indices was based on sample size limitations.

Lastly, our main conclusion from this exploratory work is that menstruation-related absenteeism and early departure are caused primarily by socio-cultural constraints, while decreased participation/concentration are caused by material constraints. However, biological constraints, such as cramps and pain, are significant impediments to both absenteeism and participation/concentration outcomes. These conclusions are strongly borne out in our data and conceptually plausible. Although, it should be noted that as some reasons were not intuitively applicable to all outcomes, girls were not given identical lists of potential reasons for each outcome (See girls’ questionnaire in supplementary information). Further research testing this hypothesis would benefit from the forethought to create identical yet logical lists of reasons for worsening educational outcomes. Moreover, future studies should define a wider set of questions for the indices a priori, to capture more nuance in the constraints faced by girls.

### Further research

There are several implications for policy. First, as other scholars have advocated, pain management should be considered as a dimension of menstrual management interventions. We call for program analysis on pain management as an intervention tool. We suggest the provision of analgesics, non-medicinal pain alleviating techniques, contraceptives and the integration of pain management into school and health curricula.

Second, research on menstrual-tracking should be completed to determine if it is a viable intervention for adolescent girls in low and middle-income countries. A clear majority of girls in this study experience variation in their menstrual cycle and, as a result, are unable to adequately prepare for their period. It is possible that improvements in menstrual-tracking technology could help ensure that girls are prepared for menstruation and ultimately improve their educational outcomes.

Third, interventions should specify which dimension of girls’ education they hope to improve (enrollment, attendance, or participation). However, comprehensive interventions may require both material and social components. Finally, the social dimension of menstruation and shame is complex. Girls express they would feel shame and fear sexualization or harassment as consequences of revealing they are post-menarche, but these reactions likely vary depending on the social sphere in question, i.e. the community, the family, with male peers, and with female peers.

Overall, we propose the development of interventions that simultaneously tackle all menstruation-related constraints: material, biological, informational, and social to create equitable educational environments and opportunities for young women.

## Supporting information

S1 Fig Distribution of constraint indices including informational. (EPS)

S2 Fig Effects of constraints on educational outcomes including knowledge and information. (EPS)

S3 Fig Effects of constraints on educational outcomes including original material constraint index. (EPS)

S1 File Girls’ questionnaire. (PDF)

S2 File Constraint index: Questions and scoring. (PDF)

S1 Data Girls’ data. (DTA)

S2 Data Girls’ do file. (DO)

## References

[1] Hennegan J., Shannon A., Rubli J., and Schwab K. Women’s and girls’ experiences of menstruation in low- and middle-income countries: A systematic review and qualitative metasynthesis. *PLoS Medicine*, 16(5), May 2019. ISSN 1549-1277. doi: 10.1371/journal.pmed.1002803 31095568 PMC6521998

[2] Sommer M., Caruso B., Torondel B., Warren E., Yamakoshi B., Haver J., et al. Menstrual hygiene management in schools: midway progress update on the “MHM in Ten” 2014-2024 global agenda. *Health Research and Policy Systems* 19(1):, 2021. doi: 10.1186/s12961-020-00669-8 33388085 PMC7776301

[3] Sommer M., Caruso B., Sahin M., Calderon T., Cavill S., Mahon T., et al. A Time for Global Action: Addressing Girls’ Menstrual Hygiene Management Needs in Schools. *PLOS Medicine*, 13(2):e1001962, February 2016. ISSN 1549-1676. doi: 10.1371/journal.pmed.1001962 26908274 PMC4764363

[4] Sommer M. Ideologies of sexuality, menstruation and risk: girls’ experiences of puberty and schooling in northern Tanzania. Culture, Health & Sexuality, 11(4):383–398, 2009. ISSN 13691058, 14645351. URL http://www.jstor.org/stable/27784458. 19326264 10.1080/13691050902722372

[5] Tamiru S., Mamo K., Acidria P., and Mushi R. Towards a sustainable solution for school menstrual hygiene management: cases of Ethiopia, Uganda, South-Sudan, Tanzania, and Zimbabwe. *Waterlines*, 34(1):92–102, January 2015. ISSN 0262-8104. doi: 10.3362/1756-3488.2015.009

[6] Tellier, S., and Hyttel, M. Menstrual Health Management in East and Southern Africa: a Review Paper. *UNFPA ESARO*, page 52, May 2018.

[7] Mason L., Nyothach E., Alexander K., Odhiambo F., Eleveld A., Vulule J., et al. ’We keep it secret so no one should know’—A qualitative study to explore young schoolgirls attitudes and experiences with menstruation in rural Western Kenya. *PLoS ONE*, 8(11), 2013. ISSN 19326203. doi: 10.1371/journal.pone.0079132 PMC3828248 24244435

[8] Adinma E., and Adinma J. Perceptions and Practices on Menstruation amongst Nigerian Secondary School Girls. *African Journal of Reproductive Health / La Revue Africaine de la Santé Reproductive*, 12(1):74–83, April 2008. ISSN 1118-4841. 20695158

[9] Oruko K., Nyothach E., Zielinski-Gutierrez E., Mason L., Alexander K., Vulule J., et al. ’He is the one who is providing you with everything so whatever he says is what you do’: A Qualitative Study on Factors Affecting Secondary Schoolgirls’ Dropout in Rural Western Kenya. *PLoS ONE*, 10(12), December 2015. ISSN 1932-6203. doi: 10.1371/journal.pone.0144321 26636771 PMC4670214

[10] Plesons M., Patkar A., Babb J., Balapitiya A., Carson F., Caruso B., et al. The state of adolescent menstrual health in low- and middle-income countries and suggestions for future action and research. Reproductive Health, 18(1): 31, 2021. URL 10.1186/s12978-021-01082-2 33557877 PMC7869499

[11] UNICEF. Guidance on Menstrual Health and Hygiene. *UNICEF*, 2019. URL https://www.unicef.org/media/91341/file/UNICEF-Guidance-menstrual-health-hygiene-2019.pdf.

[12] Sommer M. Where the education system and women’s bodies collide: The social and health impact of girls’ experiences of menstruation and schooling in Tanzania. *Journal of Adolescence*, 33(4):521–529, 2010. ISSN 0140-1971. doi: 10.1016/j.adolescence.2009.03.008 19395018

[13] Grant M., Lloyd C., and Mensch B. Menstruation and School Absenteeism: Evidence from Rural Malawi. *Comparative Education Review*, 57(2):260–284, May 2013. ISSN 0010-4086. doi: 10.1086/669121 25580018 PMC4286891

[14] Tegegne T., and Sisay M. Menstrual hygiene management and school absenteeism among female adolescent students in Northeast Ethiopia. *BMC public health*, 14(1):1118, 2014. ISSN 1471-2458. doi: 10.1186/1471-2458-14-1118 25355406 PMC4232635

[15] Miiro G., Rutakumwa R., Nakiyingi-Miiro J., Nakuya K., Musoke S., Namakula J., et al. Menstrual health and school absenteeism among adolescent girls in Uganda (MENISCUS): a feasibility study. *BMC Women’s Health*, 18(1):1–13, January 2018. ISSN 1472-6874. doi: 10.1186/s12905-017-0499-3 29298699 PMC5753466

[16] Muthengi, E., and Austrian, K. Cluster randomized evaluation of the Nia Project: study protocol. *Reproductive Health; London*, 15, 2018. http://dx.doi.org.ezproxy.cul.columbia.edu/10.1186/s12978-018-0586-4. URL http://search.proquest.com/docview/2168620161/abstract/8410A34B495B43E9PQ/1. 10.1186/s12978-018-0586-4 PMC6310925 30594217

[17] van Eijk A., Laserson K., Nyothach E., Oruko K., Omoto J., Mason L., et al. Use of menstrual cups among school girls: longitudinal observations nested in a randomised controlled feasibility studyin in rural western Kenya. *Reproductive Health*. 15(139):, August 2018. 10.1186/s12978-018-0582-8. PMC6098596 30119636

[18] Phillips-Howard P., Nyothach E., ter Kuile F., and Omoto J. Menstrual cups and sanitary pads to reduce school attrition, and sexually transmitted and reproductive tract infections: a cluster randomised controlled feasibility study in rural Western Kenya. *BMJ open*, 6(11), 2016. ISSN 2044-6055. doi: 10.1136/bmjopen-2016-013229 27881530 PMC5168542

[19] Oster E., and Thornton R. Menstruation, sanitary products, and school attendance. *American economic journal. Applied economics*, 3(1):91–100, 2011. ISSN 1945-7790. doi: 10.1257/app.3.1.91

[20] Benshaul-Tolonen, A., Zulaika, G., Nyothach, E., Odour, C., Mason, L., Obor, D., et al. Sanitary products, absenteeism and psychosocial well-being: Evidence from a three-arm cluster randomized controlled feasibility study in Western Kenya *Columbia Center for Development Economics and Policy*, Working Paper 93, March 2021.

[21] Montgomery P., Hennegan J., Dolan C., and Wu M. Menstruation and the Cycle of Poverty: A Cluster Quasi-Randomised Control Trial of Sanitary Pad and Puberty Education Provision in Uganda. *PloS one*, 11(12):e0166122, 2016. ISSN 1932-6203. doi: 10.1371/journal.pone.0166122 28002415 PMC5176162

[22] Hennegan J., and Sol L. Confidence to manage menstruation at home and at school: Findings from a cross-sectional survey of schoolgirls in rural Bangladesh. *Culture*, *Health*, *and Sexuality*, 22(2):146–165, 2020. URL 10.1080/13691058.2019.1580768. 30931818

[23] Kansiime C., Hytti L., Nalugya R., Nakuya K., Namirembe P., Nakalema S., et al. Menstrual health intervention and school attendance in Uganda (MENISCUS-2): a pilot intervention study. *BMJ Open*, 10(2):e031182, February 2020. ISSN 2044-6055, 2044-6055. doi: 10.1136/bmjopen-2019-031182 32024786 PMC7044877

[24] Freeman M., Greene L., Dreibelbis R., and Saboori S. Assessing the impact of a school based water treatment, hygiene and sanitation programme on pupil absence in Nyanza Province, Kenya: a cluster randomized trial. *Tropical Medicine & International Health*, 17(3):380–391, March 2012. ISSN 1360-2276. doi: 10.1111/j.1365-3156.2011.02927.x 22175695

[25] Benshaul-Tolonen A., Zulaika G., Sommer M., and Phillips-Howard P. Measuring Menstruation-Related Absenteeism Among Adolescents in Low-Income Countries. *The Palgrave Handbook of Critical Menstruation Studies* 705–723, 2020. 33347214

[26] Malhotra A., Goli S., Coates S., Mosquera-Vasquez M. Factors associated with knowledge, attitudes, and hygiene practices during menstruation among adolescent girls in Uttar Pradesh. Waterlines, 35(3): 277–205, 2016. URL http://www.jstor.org/stable/26600766

[27] Ndlovu E., and Bhala E. Menstrual hygiene—A salient hazard in rural schools: A case of Masvingo district of Zimbabwe. *Jamba (Potchefstroom*, *South Africa)*, 8(2):204, 2016. ISSN 1996-1421. doi: 10.4102/jamba.v8i2.204 29955312 PMC6014141

[28] McMahon S., Winch P., Caruso B., Ogutu E., Ochari I., and Rheingans R. ‘The girl with her period is the one to hang her head’ Reflections on menstrual management among schoolgirls in rural Kenya. BMC International Health and Human Rights, 11(1):1–10, 2011. ISSN 1472698X. doi: 10.1186/1472-698X-11-7 21679414 PMC3129305

[29] Sivakami M., van Eijk A., Thakur H., Kakade N., Patil C., Shinde S., et al. Effect of menstruation on girls and their schooling, and facilitators of menstrual hygiene management in schools: surveys in government schools in three states in India, 2015. *Journal of Global Health*, 9(1), June 2019. ISSN 2047-2978. doi: 10.7189/jogh.09.010408 30546869 PMC6286883

[30] Mohammed S., Larsen-Reindorf R., and Awal I. Menstrual Hygiene Management and School Absenteeism among Adolescents in Ghana: Results from a School-Based Cross-Sectional Study in a Rural Community. *International Journal of Reproductive Medicine*, April 2020. 10.1155/2020/6872491. URL https://www.hindawi.com/journals/ijrmed/2020/6872491/. ISSN: 2356-7104 Library Catalog: www.hindawi.com Pages: e6872491 Publisher: Hindawi Volume: 2020. 32411782 PMC7204135

[31] Vashisht A., Pathak R., Agarwalla R., Patavegar B., and Panda M. School absenteeism during menstruation amongst adolescent girls in Delhi, India. *Journal of Family & Community Medicine*, 25(3):163–168, 2018. ISSN 1319-1683. doi: 10.4103/jfcm.JFCM_161_17 30220845 PMC6130156

[32] Mahon T., and Fernandes M. Menstrual hygiene in South Asia: a neglected issue for WASH (water, sanitation and hygiene) programmes. Gender and Development, 18(1):99–113, 2010. ISSN 1355-2074. URL https://www.jstor.org/stable/25758884. Publisher: [Taylor & Francis, Ltd., Oxfam GB]. doi: 10.1080/13552071003600083

[33] Das P., Baker K., Dutta A., Swain T., Sahoo S., Das B., et al. Menstrual Hygiene Practices, WASH Access and the Risk of Urogenital Infection in Women from Odisha, India. *PLOS ONE*, 10(6):e0130777, June 2015. ISSN 1932-6203. doi: 10.1371/journal.pone.0130777 26125184 PMC4488331

[34] Fakhri M., Hamzehgardeshi Z., Hajikhani Golchin N., and Komili A. Promoting menstrual health among persian adolescent girls from low socioeconomic backgrounds: a quasi-experimental study. *BMC Public Health*, 12(1):193, March 2012. ISSN 1471-2458. doi: 10.1186/1471-2458-12-193 22420743 PMC3348061

[35] Fetohy E. Impact of a health education program for secondary school Saudi girls about menstruation at Riyadh city. *The Journal of the Egyptian Public Health Association*, 82(1-2):105–126, 2007. ISSN 0013-2446. 18217327

[36] Tuli, A., Chopra, S., Kumar, N., and Singh, P. Learning *from* and *with* Menstrupedia: Towards Menstrual Health Edcuation in India *Proceedings of the ACM on Human-Computer Interaction* 2, Article 174, November 2018.

[37] Benshaul-Tolonen A., Zulaika G., Nyothach E., Mason L., Obor D., Alexander K., et al. Pupil Absenteeism, Measurement, and Menstruation: Evidence from Western Kenya. (No. 74):57, March 2019.

[38] Rheinländer T., Gyapong M., Akpakli D., and Konradsen F. Secrets, shame and discipline: School girls’ experiences of sanitation and menstrual hygiene management in a peri-urban community in Ghana. *Health Care for Women International*, 40(1):13–32, January 2019. ISSN 0739-9332. doi: 10.1080/07399332.2018.1444041 29485336

[39] Benshaul-Tolonen A., Aguilar-Gomez S., Heller Batzer N., Cai R., Charles Nyanza E., Period teasing, stigma and knowledge: A survey of adolescent boys and girls in Northern Tanzania. *PlosOne* 15(10):1–21, October 2020. doi: 10.1371/journal.pone.0239914 33112868 PMC7592731

[40] Sahin M., Tamiru S., Mamo K., Acidria P., Mushi R., Ali C., et al. Towards a sustainable solution for school menstrual hygiene management: cases of Ethiopia, Uganda, South-Sudan, Tanzania, and Zimbabwe. *Waterlines*, 34(1):92–102, January 2015. ISSN 0262-8104. doi: 10.3362/1756-3488.2015.009

[41] Mukherjee A., Lama M., Khakurel U., Jha A., Ajose F., Acharya S., et al. Perception and practices of menstruation restrictions among urban adolescent girls and women in Nepal: a cross-sectional survey. *Reproductive Health*, 17, June 2020. ISSN 1742-4755. doi: 10.1186/s12978-020-00935-6 32487096 PMC7268527

[42] Chandra-Mouli V., and Patel S. Mapping the knowledge and understanding of menarche, menstrual hygiene and menstrual health among adolescent girls in low- and middle-income countries. *Reproductive Health*, 14(1), December 2017. ISSN 1742-4755. doi: 10.1186/s12978-017-0293-6 28249610 PMC5333382

[43] Gade, A., and Hytti, L. Menstrual health in Rhino Camp refugee settlement, West Nile, Uganda—Resources • SuSanA. Pilot project intervention report., WoMena Uganda and ZOA, 2017. URL https://www.susana.org/en/knowledge-hub/resources-and-publications/library/details/3506. Library Catalog: www.susana.org.

[44] Ibitoye M., Choi C., Tai H., Lee G., and Sommer M. Early menarche: A systematic review of its effect on sexual and reproductive health in low- and middle-income countries. *PloS One*, 12(6):e0178884, 2017. ISSN 1932-6203. doi: 10.1371/journal.pone.0178884 28591132 PMC5462398

[45] De Sanctis V., Soliman A., Elsedfy H., Soliman A., Soliman R., and El Kholy M. Dysmenorrhea in adolescents and young adults: a review in different country. *Acta Bio-Medica: Atenei Parmensis*, 87(3):233–246, 2016. ISSN 2531-6745. 28112688 PMC10521891

[46] Ministry of Health Uganda. Adolescent Health Risk Behaviors in Uganda: A National Cross Sectional Study report 2017 | Ministry of Health Knowledge Management Portal. Technical report, UNICEF, WHO, UN WOMEN, UNFPA, UNAIDS, Uganda, 2016. URL http://library.health.go.ug/publications/adolescent-health/adolescent-health-risk-behaviors-uganda-national-cross-sectional.

[47] Hennegan J., Dolan C., Wu M., Scott L., and Montgomery P. Measuring the prevalence and impact of poor menstrual hygiene management: a quantitative survey of schoolgirls in rural uganda. *BMJ Open*, 6(12), 2016. ISSN 2044-6055. doi: 10.1136/bmjopen-2016-012596 28039290 PMC5223625

[48] Kothari B. Perception about Menstruation: A Study of Rural Jaipur, Rajasthan. Indian Anthropologist, 40(1):43–54, 2010. ISSN 09700927. URL http://www.jstor.org/stable/41920109.

[49] Mason L., Nyotach E., van Eijk A., Obor D., Alexander K., Ngere I., et al. Comparing use and acceptability of menstrual cups and sanitary pads by schoolgirls in rural Western Kenya. *International Journal of Reproduction*, *Contraception*, *Obstetrics and Gynecology*, 8(8):2974–2982, August 2019. 10.18203/2320-1770.ijrcog20193506

[50] Caruso, B., Fehr, A., Inden, K., Sahin, M., Ellis, A., Andes, KL., et al. WASH in Schools Empowers Girls’ Education in Freetown, Sierra Leone: An Assessment of Menstrual Hygiene Management in Schools *United Nations Children’s Fund*, 2013. URL https://www.washinschoolsindex.com/storage/articles/GOU8xvmngOFdmlr4UxSDSQq2m64tlaZwpqTRREm4.pdf

[51] Caruso B., Cooper H., Haardörfer R., Yount K., Routray P., Torondel B., et al. The association between women’s sanitation experiences and mental health: A cross-sectional study in rural, odisha india. *SSM—Population Health*, 5:257–266, 2018. ISSN 2352-8273. URL http://www.sciencedirect.com/science/article/pii/S2352827318300521 30094321 10.1016/j.ssmph.2018.06.005 PMC6077264

[52] Phillips-Howard P., Otieno G., Burmen B., Otieno F., Odongo F., Odour C., et al. Menstrual Needs and Associations with Sexual and Reproductive Risks in Rural Kenyan Females: A Cross-Sectional Behavioral Survey Linked with HIV Prevalence. *Journal of Women’s Health*, 24(10):801–811, October 2015. ISSN 1540-9996. doi: 10.1089/jwh.2014.5031 26296186 PMC4624246

[53] Montgomery P., Ryus C., Dolan C., Dopson S., Scott L., Sanitary pad interventions for girls’ education in Ghana: A pilot study *PloS one*, 7(10):, 2012. doi: 10.1371/journal.pone.0048274 23118968 PMC3485220

[54] Babagoli, M., Benshaul-Tolonen, A., Zulaika, G., Nyothach, E., Oduor, C., Obor, D., et al. The Cost-Benefit and Cost-effectiveness of Providing Menstrual Cups and Sanitary Pads to Schoolgirls in Rural Kenya. *Columbia Center for Development Economics and Policy*, *Working Paper* 87, June 2020.

[55] Caruso B., Freeman M., Garn J., Dreibelbis R., Saboori S., Muga R., et al. Assessing the impact of a school based latrine cleaning and handwashing program on pupil absence in Nyanza Province, Kenya: a cluster randomized trial. *Tropical medicine & international health: TM & IH*, 19(10):1185–1197, October 2014. ISSN 1365-3156. doi: 10.1111/tmi.12360 25055716 PMC4876949

[56] Loaiza, E., and Lloyd, C. Adolescents and Education in Africa. Discussion summary, UNICEF, New York, 2008.

[57] Rebacz E. Age at menarche in schoolgirls from Tanzania in light of socioeconomic and sociodemographic conditioning. *Collegium Antropologicum*, 33(1):23–29, March 2009. ISSN 0350-6134. 19408599

[58] Emdadul Haque S., Rhman M., Itsuko K., Mutahara M., and Sakisaka K. The effect of a school-based educational intervention on menstrual health: an intervention study among adolescent girls in Bangladesh *BMJ Open* 4(7):, 2014. 10.1136/bmjopen-2013-004607 PMC4091465 24993753

[59] Coast E., Lattof S., and Strong J. Puberty and menstruation knowledge among young adolescents in low-and middle-income countries:a scoping review. *International Journal of Public Health*, 64(2):293–304, 2019. doi: 10.1007/s00038-019-01209-0 30740629 PMC6439145

[60] World Bank. GDP per capita (current US$)-Tanzania. *World Development Indicators*, *The World Bank Group* URL https://data.worldbank.org/indicator/NY.GDP.PCAP.CD?locations=TZ.

[61] World Bank. The World Bank in Tanzania Overview *The World Bank*, March 2021. URL https://www.worldbank.org/en/country/tanzania/overview.

[62] Swinkels, R. Poverty and Equality Brief: Tanzania *The World Bank*, April 2020. URL https://databank.worldbank.org/data/download/poverty/33EF03BB-9722-4AE2-ABC7-AA2972D68AFE/Global_POVEQ_TZA.pdf.

[63] Chinyama J., Chipungu J., Rudd C., Mwale M., Verstraete L., Sikamo C., et al., Menstrual hygiene management in rural schools of zambia: A descriptive study of knowledge, experiences and challenges faced by schoolgirls. *BMC Public Health*, 19, 12 2019. doi: 10.1186/s12889-018-6360-2 30611223 PMC6321718

[64] Girod C., Ellis A., Andes K., and Freeman M. Physical, Social, and Political Inequities Constraining Girls’ Menstrual Management at Schools in Informal Settlements of Nairobi, Kenya. *Journal of Urban Health*, 94(6):835–846, December 2017. ISSN 1099-3460. doi: 10.1007/s11524-017-0189-3 28875308 PMC5722726

[65] Jain M., and Yammiyavar P. Game Based Learing Tool Seeking Peer Support for Empowering Adolescent Girls in Rural Assam. *International Conference on Interaction Design and Children*, 275–278, June 2015.

[66] Connolly S., and Sommer M. Cambodian Girls’ Recommendations for Facilitating Menstrual Hygiene Management in School *Journal of Water*, *Sanitation and Hygiene for Development*, 3(4):, 612–622, 2013. doi: 10.2166/washdev.2013.168

[67] Kuhlmann A., Henry K., and Wall L. Menstrual Hygiene Management in Resource-Poor Countries. *Obstetrical & Gynecological Survey*, 72(6):356–376, June 2017. ISSN 0029-7828. doi: 10.1097/OGX.0000000000000443 28661550 PMC5482567

[68] WIlliams C.E., and Creighton S.M. Menstrual Disorders in Adolescents: Review of Current Practice. *Hormone Research in Paediatrics*, 78(3):135–43, 2012. doi: 10.1159/000342822 23051587

[69] Ray S., Kumar Mishra S., Ghosh Roy A., and Mohan Das B. Menstrual Characteristics: A study of the Adolescents of rural and urban West Bengal, India *Annals of Human Biology* 37(5):668–81, 2010. doi: 10.3109/03014460903563442 20166852

[70] Epstein, D., Lee, N., Kang, J., Agapie, E., Schroeder, J., Pina, L., et al. Examining Menstrual Tracking to Inform the Design of Personal Informatics Tools. *Proceedings of the SIGCHI conference on human factors in computing systems.CHI Conference*, 6876—6888, 2017. 10.1145/3025453.3025635 PMC5432133 28516176

[71] Levy J., and Romo-Aviles N. A good little tool to get to know yourself a bit better: a qualitative study on user’s experiences of app-supported menstruation tracking in Europe. *BMC Public Health* 19(1):1213, 2019. doi: 10.1186/s12889-019-7549-8 31481043 PMC6724299

[72] Fox S., and Epstein D. Monitoring Menses: Design Based Investigations of Menstrual Tracking Applications *The Palgrave Handbook of Critical Menstruation Studies* 733–750, 2020. doi: 10.1007/978-981-15-0614-7_54 33347158

[73] Moglia M., Nguyen H., Chyjek K., Chen K., and Castano P. Evaluations of Smartphone Menstrual Cycle Tracking Applications Using an Adapted APPLICATIONS Scoring System. *Obstetrics and Gynecology*, 127(6):, June 2016. doi: 10.1097/AOG.0000000000001444 27159760

[74] Jacobson A., Vesely S., Haamid F., Christian-Rancy M., and O’Brien S. Mobile Application vs Paper Pictorial Blood Assessment Chart to Track Menses in Young Women: A Randomized Cross-Over Design. *J Pediatr Adolesc Gynecol.*, 31(2):, 84–88, April 2018. doi: 10.1016/j.jpag.2017.09.009 29030160

[75] Houghton L.E., Howland R., and McDonald J. Mobilizing Breast Cancer Prevention Research Through Smartphone Apps: A Systematic Review of the Literature. *Front Public Health*, 7, 298, November 2019. doi: 10.3389/fpubh.2019.00298 31781525 PMC6851054

[76] Austrian, K., Kangwana, B., Muthengi, E., and Soler-Hampejsek, E. Effects of Sanitary Pad Distribution and Reproductive Health Education on Primary School Attendance and Reproductive Health Knowledge and Attitudes in Kenya: A Cluster Randomized Controlled Trial. *Working Paper*, November 2020. 10.21203/rs.3.rs-105989/v1 PMC8406733 34465344

[77] Dreibelbis R., Greene L., Freeman M., Saboori S., Chase R., and Rheingans R. Water, sanitation, and primary school attendance: A multi-level assessment of determinants of household-reported absence in Kenya. *International Journal Of Educational Development* 33(5):, 457–465, 2013. doi: 10.1016/j.ijedudev.2012.07.002

[78] Dolan C., Ryus C., Dopson S., and Montgomery P. A blind spot in girls’ education: Menarche and its webs of exclusion in Ghana. *Journal of International Development*, 26(5):643–657, 2014. ISSN 0954-1748. doi: 10.1002/jid.2917

[79] Miguel E. Evidence on Research Transparency in Economics. Journal of Economic Perspectives 35(2):, 193–214, 2021. doi: 10.1257/jep.35.3.193

