# Supplementary material for: Biological, material and socio-cultural constraints to effective menstrual hygiene management among secondary school students in Tanzania
Source: PLOS Glob Public Health. 2022 Mar 14;2(3):e0000110. doi: 10.1371/journal.pgph.0000110 (PMC10021794; doi:10.1371/journal.pgph.0000110)
Supplement: S1 File — (PDF) [file pgph.0000110.s004.pdf]

**SUPPLEMENTARY MATIERAL**

Surveying date:
School ID:
Classroom ID:
Student ID:

---

# GIRLS QUESTIONNAIRE

**Dodoso**

**Kipengele 1: Marafiki**

1. Tungependa utuambie kuhusu marafiki zako 5 wa KIKE wa karibu kabisa ulionao shuleni. Tafadhali taja majina ya marafiki zako wa karibu kabisa. Taja tu marafiki ambao wanasoma shule hii, na ambao wako darasa moja na wewe, na ambao unawahesabu kama marafiki wa karibu.

*Andika majina kamili na darasa la marafiki uliowataja*

**a.** ______________________________
**b.** ______________________________
**c.** ______________________________
**d.** ______________________________
**e.** ______________________________

2. Sasa tungependa utuambie kuhusu marafiki wa KIUME wa karibu sana ulio nao shuleni. Tafadhali yataje majina ya marafiki zako wa karibu kabisa. Taja tu marafiki ambao wanasoma shule hii, na ambao wako darasa moja na wewe, na ambao unawahesabu kama marafiki wa karibu.

*Andika majina kamili na darasa la marafiki uliowataja*

**a.** ______________________________
**b.** ______________________________
**c.** ______________________________
**d.** ______________________________
**e.** ______________________________

**Kipengele 2: Taarifa**
*Tungependa kukuuliza maswali kadhaa kuhusu hedhi.*

1. Kwanini msichana au mwanamke anapata hedhi? Chagua yote yanayokubalika.
   - ☐ dhambi binafsi
   - ☐ kutokana na nguvu za giza
   - ☐ kwa sababu hakupata mimba hedhi huo
   - ☐ kutoa damu mbaya
   - ☐ mengineyo
   - ☐ sijui

2. Ni mara ngapi msichana (ambaye tayari ameshapata hedhi) anapata hedhi kwa wastani?
   - ☐ mara moja katika maisha yake
   - ☐ mara moja kila mwaka
   - ☐ kila wiki
   - ☐ kila hedhi
   - ☐ sijui

Surveying date:
School ID:
Classroom ID:
Student ID:

---

3. Kwa wastani hedhi huwa inachukua muda gani? Chagua moja.
   - [ ] saa moja
   - [ ] siku moja
   - [ ] siku kadhaa
   - [ ] wiki mbili
   - [ ] muda wote
   - [ ] sijui

4. Kiujumla ni katika umri gani wasichana huwa wanapata hedhi yao kwa mara ya kwanza?

   ___________________________________________________________________________

5. Katika umri gani wanawake hufikia ukomo na kupata hedhi?

   ___________________________________________________________________________

*6.* Chagua jibu sahihi
*Tafadhali chagua Ndio au Hapana kuashiria kama kauli zifuatazo ni za ukweli.*

a) Msichana akivunja ungo (kupata hedhi kwa mara ya kwanza), inamaanisha anaweza kupata mimba akifanya mapenzi.
   ☐ Ndio ☐ Hapana ☐ Sijui
b) Damu ya kipindi cha hedhi hutokana na kumwaga damu ya ukuta ndani ya tumbo la uzazi
   ☐ Ndio ☐ Hapana ☐ Sijui
c) Hedhi huashiria kwamba mwanamke hana mimba
   ☐ Ndio ☐ Hapana ☐ Sijui
d) Kupevuka mayai hutokea kwa wastani siku 14 kabla ya hedhi
   ☐ Ndio ☐ Hapana ☐ Sijui
e) Kupevuka mayai hutokea kipindi cha hedhi
   ☐ Ndio ☐ Hapana ☐ Sijui
f) Je, huwa ni kawaida kwa wasichana kujisikia maumivu ya kimwili wakipata hedhi?
   ☐ Ndio ☐ Hapana ☐ Sijui
g) Je, wasichana wengi hupitia yafuatayo wakiwa hedhini?

| | | | |
|---|---|---|---|
| Hisia mbaya zisizozuilika [hasira] | ☐ Ndio | ☐ Hapana | ☐ Sijui |
| Mfadhaiko | ☐ Ndio | ☐ Hapana | ☐ Sijui |
| Uchovu mkali | ☐ Ndio | ☐ Hapana | ☐ Sijui |

**Kipengele 3: Uzoefu Binafsi**

1. Kuna mtu yeyote amewahi kukuambia kuhusu hedhi na kwanini inatokea?
   ☐ NDIO ☐ HAPANA

   Kama ndio, nani? Tafadhali chagua zote zinazokubalika.

| | |
|---|---|
| ☐ mama | ☐ baba |
| ☐ bibi | ☐ babu |
| ☐ dada | ☐ kaka |

**SUPPLEMENTARY MATIERAL**

Surveying date:
School ID:
Classroom ID:
Student ID:

---

| | |
|---|---|
| ☐ Rafiki wa kike | ☐ rafiki wa kiume |
| ☐ shangazi au ndugu wa kike | ☐ mjomba au ndugu wa kiume |
| ☐ nesi | ☐ mchungaji / kiongozi wa kidini |
| ☐ mwalimu wa kike | ☐ mwalimu wa kiume |
| ☐ mpenzi/mwenza | ☐ mwingine |

2. Uliwahi kupokea taarifa kuhusu hedhi kutoka kwa wafuatao:

| | | |
|---|---|---|
| • Mtandaoni | ☐ NDIO | ☐ HAPANA |
| • Mtoa huduma ya afya | ☐ NDIO | ☐ HAPANA |
| • Machapisho ya shuleni (vitabu, madarasa, homework, n.k.) | ☐ NDIO | ☐ HAPANA |
| • Vipeperushi (matangazo, mabango) | ☐ NDIO | ☐ HAPANA |

3. Ulifanya nini uliposikia kuhusu hedhi mara ya kwanza? (Tafadhali chagua moja kutoka kila kundi la hizo 3, ambapo 2 ni katikati.)

| | 1 | 2 | 3 | |
|---|---|---|---|---|
| ulishangaa | ☐ | ☐ | ☐ | sikushangaa |
| uliogopa | ☐ | ☐ | ☐ | sikuogopa |
| ulipata msisimko | ☐ | ☐ | ☐ | hukusisimka |
| ulichanganyikiwa | ☐ | ☐ | ☐ | hukuchanganyikiwa |
| ulijisikia ujasiri | ☐ | ☐ | ☐ | hukujisikia jasiri |
| ulisikia kinyaa | ☐ | ☐ | ☐ | hukusikia kinyaa |
| ulijivunia | ☐ | ☐ | ☐ | hukujivunia |
| ulijionea haya | ☐ | ☐ | ☐ | hukujionea haya |

4. Kuna mtu amewahi kukuambia nini unapaswa kufanya ukianza kupata hedhi?
☐ NDIO ☐ HAPANA

Kama ndio, ulipokea ushauri gani? Tafadhali chagua yote yanayokubalika.
☐ namna ya kutumia taulo/vitambaa/vitambaa vya pamba
☐ namna ya kubakia msafi/kunawa
☐ namna ya kumudu mauimivu
☐ mambo/shughuli za kuepuka wakati wa hedhi

---

☐ mengineyo ___________

5. Weka alama kwa watu wote ambao UNGEPENDA kuongea nao kuhusu hedhi yako.

| | |
|---|---|
| ☐ mama | ☐ baba |
| ☐ bibi | ☐ babu |
| ☐ dada | ☐ kaka |
| ☐ rafiki wa kike | ☐ rafiki wa kiume |
| ☐ shangazi au ndugu mwingine wa kike | ☐ mjomba au ndugu wa kiume |
| ☐ nesi | ☐ mchungaji / kiongozi wa kidini |
| ☐ mwalimu wa kike | ☐ mwalimu wa kiume |
| ☐ mpenzi/mwenza | ☐ mwingine |
| ☐ hakuna hata mtu mmoja | |

6. Na nani USINGEPENDA KABISA kuongea naye kuhusu hedhi yako. Tafadhali chagua yote yanayokubalika.

| | |
|---|---|
| ☐ mama | ☐ baba |
| ☐ bibi | ☐ babu |
| ☐ dada | ☐ kaka |
| ☐ rafiki wa kike | ☐ rafiki wa kiume |
| ☐ shangazi au ndugu mwingine wa kike | ☐ mjomba au ndugu wa kiume |
| ☐ nesi | ☐ mchungaji / kiongozi wa kidini |
| ☐ mwalimu wa kike | ☐ mwalimu wa kiume |
| ☐ mpenzi/mwenza | ☐ mwingine |
| ☐ hakuna hata mtu mmoja | |

7. Umeshapata hedhi yako?
   ☐ Ndio **(endelea swali linalofuata)**    ☐ Hapana **(nenda Kipengele 4, Swali 1)**

8. Katika umri gani ulipata hedhi mara ya kwanza? _______

9. Je, ulipopata hedhi mara ya kwanza ilikuwa kitu cha hali ya chanya, hasi, au hali tu ya kawaida?
   ☐ chanya    ☐ hasi    ☐ katikati

10. Ulijisikiaje ulipopata hedhi kwa mara ya kwanza? Ulikuwa…
(Tafadhali chagua moja kutoka kila kundi la hizo 3, ambapo 2 ni katikati.)

| | 1 | 2 | 3 | |
|---|---|---|---|---|
| 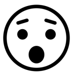 ulishangaa | ☐ | ☐ | ☐ | sikushangaa |
| 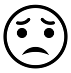 uliogopa | ☐ | ☐ | ☐ | sikuogopa |
| 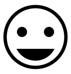 ulipata msisimko | ☐ | ☐ | ☐ | hukusisimka |

---

| | | | | | |
|---|---|---|---|---|---|
| 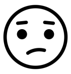 | ulichanganyikiwa | ☐ | ☐ | ☐ | hukuchanganyikiwa |
| 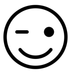 | ulijisikia ujasiri | ☐ | ☐ | ☐ | hukujisikia jasiri |
| 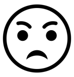 | ulisikia kinyaa | ☐ | ☐ | ☐ | hukusikia kinyaa |
| 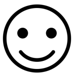 | ulijivunia | ☐ | ☐ | ☐ | hukujivunia |
| 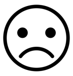 | ulijionea haya | ☐ | ☐ | ☐ | hukujionea haya |

11. Fikiria nyuma hadi muda kabla hujapata hedhi. Mtu yeyote alikuambia kuhusu hedhi kabla haijaanza?

    ☐ NDIO ☐ HAPANA

Kama ndio, nani? Tafadhali chagua zote zinazokubali.

| | |
|---|---|
| ☐ mama | ☐ baba |
| ☐ bibi | ☐ babu |
| ☐ dada | ☐ kaka |
| ☐ rafiki wa kike | ☐ rafiki wa kiume |
| ☐ shangazi au ndugu mwingine wa kike | ☐ mjomba au ndugu wa kiume |
| ☐ nesi | ☐ mchungaji / kiongozi wa kidini |
| ☐ mwalimu wa kike | ☐ mwalimu wa kiume |
| ☐ mpenzi/mwenza | ☐ mwingine |

12. Hedhi yako huwa inadumu kwa siku ngapi/Hedhi yako huwa inachukua muda gani kuisha?
    siku ____________

13. Kwenye kipimo cha 1 hadi 5 ambapo 1 ni hakuna maumivu na 5 ni maumivu makali sana; Huwa unapata maumivu ya kimwili kiasi gani ukiwa kwenye hedhi?

| | **1** | **2** | **3** | **4** | **5** | |
|---|---|---|---|---|---|---|
| *Sipati maumivu kabisa* | ☐ | ☐ | ☐ | ☐ | ☐ | *Maumivu makali sana* |

14. Kwenye kipimo cha 1 hadi 5 ambapo 1 ni kutokwa damu kidogo na 5 ni kutokwa damu nyingi; Unatoka damu kiasi gani ukiwa hedhini?

| | **1** | **2** | **3** | **4** | **5** | |
|---|---|---|---|---|---|---|
| *Natokwa damu kidogo* | ☐ | ☐ | ☐ | ☐ | ☐ | *Natokwa damu nyingi* |

15. Kwenye kipimo cha 1 hadi 5, ambapo 1 ni maumivu machache na 5 ni maumivu zaidi na 3 ni sawa; Unalinganisha vipi maumivu ambayo wewe unajisikia ukipata hedhi na ambayo wanayapata wasichana wa rika lako?

| | **1** | **2** | **3** | **4** | **5** | |
|---|---|---|---|---|---|---|
| *Ninapata maumivu kidogo kuliko wao* | ☐ | ☐ | ☐ | ☐ | ☐ | *Ninapata maumivu mengi zaidi* |

Surveying date:
School ID:
Classroom ID:
Student ID:

---

16. Kwenye kipimo cha 1 hadi 5 ambapo 1 ni kutokwa damu kidogo zaidi na 5 ni damu nyingi zaidi na 3 ni sawa; Unafikiri wewe unatokwa damu nyingi zaidi, kidogo zaidi, au sawa na wenzako?

| | 1 | 2 | 3 | 4 | 5 | |
|---|---|---|---|---|---|---|
| *Ninatokwa damu kidogo zaidi* | ☐ | ☐ | ☐ | ☐ | ☐ | *Ninatokwa damu nyingi zaidi* |

17. Kipindi cha hedhi kinadumu kuanzia siku ya kwanza ya hedhi (kutokwa damu) hadi siku moja kabla ya mwanzo wa kipindi kingine cha hedhi (siku ya kwanza kutokwa damu). Unajua wewe mzunguko wa hedhi yako unachukua muda gani kwa wastani? Kwa maana nyingine, huwa unatunza kumbukumbu ya kwamba unatokwa damu baada ya muda gani?
☐ NDIO ☐ HAPANA

18. Unafikiri kwamba kwako muda kati ya hedhi na hedhi unatofautiana sana?
☐ NDIO ☐ HAPANA

19. Je, inakuwia vigumu kujua kwamba utapata hedhi hivi karibuni na kujiandaa?
☐ NDIO ☐ HAPANA

20. Ukiwa hedhini, au punde tu kabla, huwa unapata…:

| | | |
|---|---|---|
| Hisia mbaya zisizozuilika [hasira] | ☐ NDIO | ☐ HAPANA |
| Mfadhaiko au woga? | ☐ NDIO | ☐ HAPANA |
| Uchovu mkali? | ☐ NDIO | ☐ HAPANA |

21. Huwa unatumia dawa za maumivu? (kama vile paracetamol) kumudu maumivu ukipata hedhi?
☐ Daima ☐ Mara moja moja ☐ Mara chache ☐ Kamwe

**Kipengele 5: Bidhaa Za Hedhi**

**SUPPLEMENTARY MATIERAL**

Surveying date:
School ID:
Classroom ID:
Student ID:

---

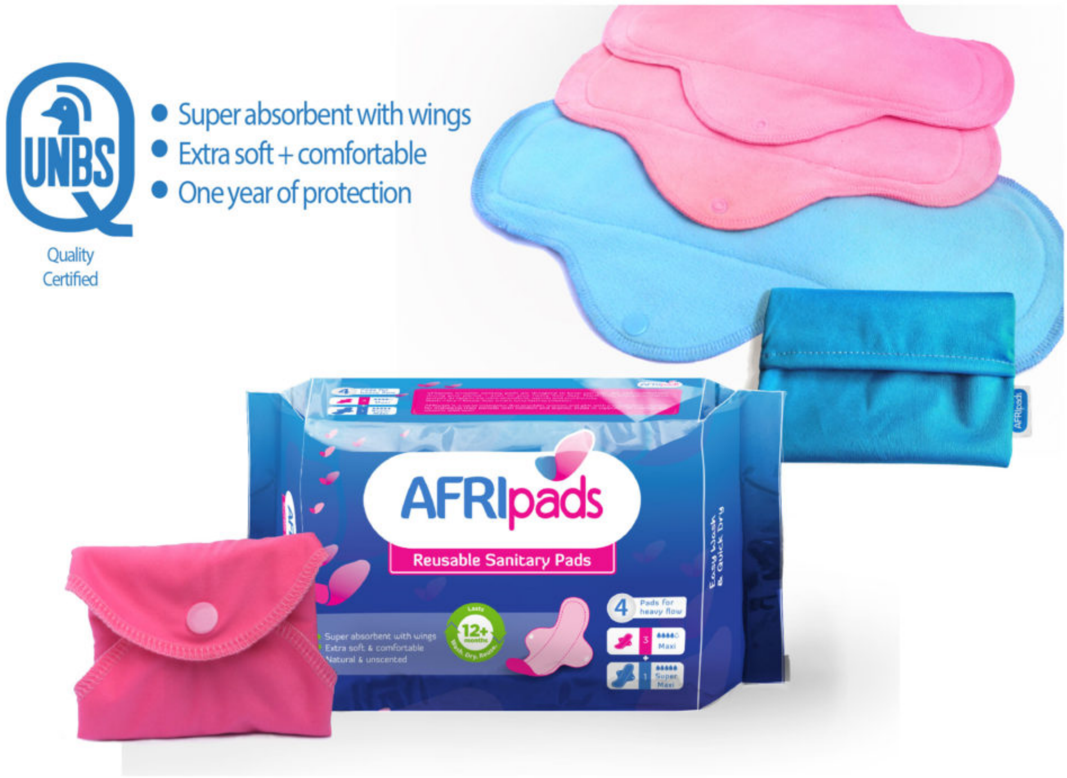


Pedi za kusafisha zinazotumika zaidi ya mara moja ni kitambaa kinachovaliwa kwenye chupi (nguo ya ndani) kuzuia damu ya hedhi kuvujia kwenye nguo. Inaweza kuoshwa, kukaushwa na kutumiwa kwa mwaka 1. Picha hapo juu inaonesha Afripads, aina mojawapo ya pedi za kutumiwa zaidi ya mara moja. Kila pakiti ina pedi 4.

1. Je umewahi Kuona ama Kusikia kuhusu pedi za kusafisha zinazotumika Zaidi ya mara moja?

    ☐ Ndio ☐ Hapana

2. Je umewahi Kutumia pedi za kusafisha zinazotumika Zaidi ya mara moja?

    ☐ Ndio ☐ Hapana

3. Huwa unatumia nini **mara moja moja** ukiwa hedhini? Tafadhali chagua **<u>yote yanayokubalika.</u>**
    ☐ kitambaa ☐ pedi
    ☐ tampuni ☐ bidhaa asilia (e.g. nyasi, majani, mavi ya ng'ombe)
    ☐ vigodoro ☐ kikombe cha hedhi
    ☐ situmii chochote ☐ nyingine __________
    ☐ pedi za kusafisha

4. Kipi huwa unakitumia **mara nyingi** ukiwa hedhini? Tafadhali chagua **<u>moja tu.</u>**
    ☐ kitambaa ☐ pedi
    ☐ tampuni ☐ bidhaa asilia (e.g. nyasi, majani, mavi ya ng'ombe)

☐ vigodoro ☐ kikombe cha hedhi
☐ situmii chochote ☐ nyingine _________
☐ pedi za kusafisha

5. Je, umewahi kutumia kitambaa?
   ☐ NDIO ☐ HAPANA

   a. Kama umewahi kutumia kitambaa, huwa unafua na sabuni vitambaa unavyotumia ukiwa hedhini?
   ☐ NDIO ☐ HAPANA ☐ Situmii kitambaa

   b. Kama huwa unatumia kitambaa, huwa unavikausha juani vitambaa ulivyovifua?
   ☐ NDIO ☐ HAPANA ☐ Situmii kitambaa
   Kama hapana, wapi ________________________________________

6. Ni njia ipi ya kujiweka safi unaipendelea zaidi? Tafadhali <u>chagua moja.</u>
   ☐ kitambaa ☐ pedi
   ☐ tampuni ☐ bidhaa asilia (e.g. nyasi, majani, mavi ya ng'ombe)
   ☐ vigodoro ☐ kikombe cha hedhi
   ☐ situmii chochote ☐ nyingine _________
   ☐ pedi za kusafisha

7. Je, daima huwa unatumia hiyo njia unayoipendelea zaidi?
   ☐ NDIO ☐ HAPANA

8. Fikiria hedhi mmoja ambapo haukutumia hiyo njia unayoipendelea zaidi. Kwanini **HAUKUTUMIA** njia unayoipendelea zaidi?
   ☐ hakuna hela ya kutosha kununua ☐ njia hiyo ilikuwa haipatikani
   ☐ kukosa ruhusa ya wazazi ☐ Sikujua jinsi ya kuitumia [kuipata?]
   ☐ niliona aibu ☐ hiyo njia haikuwa salama
   ☐ nyingine ______________________________

9. Ni mara ngapi unabadilisha kifaa cha kukuweka safi kila siku unapokuwa hedhini?
   ___________________

10. Unatupaje vifaa vya kujiweka safi **(ile uliyoichagua kwenye swali 24)**?
    ☐ Siitupi (naitumia kwa kuirudia) ☐ ndoo ya taka
    ☐ chooni ☐ naichoma
    ☐ naizika ☐ nyingine ___________________________

**Kipengele 5: Uratibu wa Hedhi kwa wanarika**

1. Tafadhali kadiria kiasi cha wanafunzi wenzako wa kike ambao tayari wamevunja ungo.

   ☐ 0% 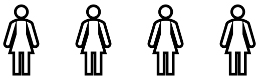

   ☐ 1-25% 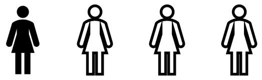

   ☐ 26-50% 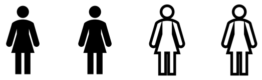

Surveying date:
School ID:
Classroom ID:
Student ID:

---

☐ 51-75%

☐ 76-100%

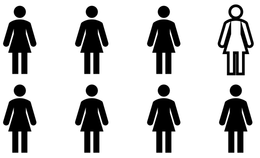

2. Mara nyingine wasichana hutaniwa kuhusu hedhi. Tafadhali kadiria kiasi cha wanafunzi wenzako wa kike ambao wamewahi kutaniwa kuhusu hedhi.

☐ 0%

☐ 1-25%

☐ 26-50%

☐ 51-75%

☐ 76-100%

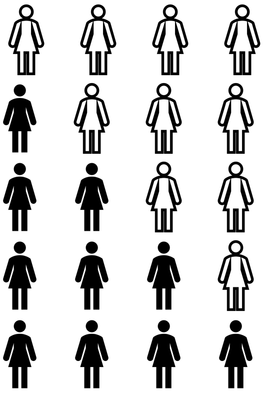

3. Tafadhali kadiria njia **inayotumika zaidi** ya kujiweka safi miongoni mwa watu wa rika lako darasani. Tafadhali chagua moja.

☐ kitambaa
☐ tampuni
☐ vigodoro
☐ situmii chochote
☐ pedi za kusafisha
☐ pedi
☐ bidhaa asilia (e.g. nyasi, majani, mavi ya ng'ombe)
☐ kikombe cha hedhi
☐ nyingine _________

4. Unajiamini kiasi gani kwamba unajua nini wanafunzi wenzio wanatumia wakiwa hedhini?

*Sijiamini kabisa* ☐1 ☐ 2 ☐ 3 ☐ 4 ☐ 5 *Najiamini kabisa*

5. Kama wanatumia pedi, unajua namna wanavyozitupa/[wanavyozitokomeza zikishatumika]?

☐ NDIO ☐ HAPANA

6. Kam ulijibu ndio kwenye swali lililopita, ni njia gani wanayoitumia zaidi?

☐ hawatupi (wanatumia zaidi ya mara moja)
☐ ndoo ya taka
☐ choo
☐ kuzichoma moto
☐ kuzizika
☐ nyingine ____________________________________________________

**Kipengele 6: Utafiti juu ya Shule**

1. Unajisikia salama kwenye vyoo vya shule?

☐ NDIO ☐ HAPANA

2. Huwa unajisikia salama kwenye vyoo vya shule ukiwa hedhini?

---

☐ NDIO ☐ HAPANA

3. Huwa unajua wakati wanafunzi wenzako wa kike wakiwa hedhini?
   ☐ NDIO ☐ HAPANA

4. Je, unahisi kama kupata hedhi ni kitu ambacho unapaswa kukificha?
   ☐ NDIO ☐ HAPANA

5. Je, ungesikia aibu kama wasichana wengine wangegundua kwamba upo hedhini?
   ☐ NDIO ☐ HAPANA

6. Je, ungesikia aibu kama wavulana wangegundua kwamba upo hedhini?
   ☐ NDIO ☐ HAPANA

7. Kama watu wangejua kwamba umewahi kupata hedhi, ungeogopa kutaniwa?
   ☐ NDIO ☐ HAPANA
   Kama ndio, nani? Tafadhali chagua yote yanayokubali.
   ☐ wasichana wa rika lako ☐ wavulana wa rika lako ☐ walimu
   ☐ hakuna yoyote ☐ mwingine ______

8. Kama watu wangejua kwamba umewahi kupata hedhi, ungeogopa kuguswa kinyume na matakwa yako, au kusisitizwa kutoka na mtu fulani?
   ☐ NDIO ☐ HAPANA
   Kama ndio, na nani? Tafadhali chagua yote yanayokubali.
   ☐ vijana wenzio ☐ walimu ☐ wanafamilia ☐ hakuna mtu
   ☐ wengine ______

   Kama ndio, una wasiwasi hii itatokea wapi?
   ☐ vyoo vya kutumiwa na umati
   ☐ vyoo vya shule
   ☐ darasani
   ☐ viwanja vya shule
   ☐ nikiwa njiani kwenda shuleni au kurudi
   ☐ nafasi za umma (kama vile viwanja vya umma)
   ☐ nyumbani
   ☐ sio popote
   ☐ nyingine _________________________

9. Kama watu wangejua kwamba umewahi kupata hedhi, ungeogopa mimba isiyopangwa au kutia aibu familia?
   ☐ NDIO ☐ HAPANA

10. Kama watu wangejua kwamba umewahi kupata hedhi, ungeogopa kwamba walimu wasingekuwa waelewa au wenye msaada?
    ☐ NDIO ☐ HAPANA

11. Kama watu wangejua kwamba umewahi kupata hedhi, ungeogopa kuhimizwa kuolewa au kuwa na mpenzi?

---

 NDIO  HAPANA

12. Unakubaliana na kauli ifuatayo: "Nyumbani kwetu, wanawake/wasichana lazima waone aibu kuongelea au kuweka wazi hedhi yao (damu, maumivu, vifaa vya kujiweka safi) mbele ya wanafamilia."
     nakubaliana sana  nakubaliana  katikati  sikubaliani  sikubaliani kabisa

13. Unakubaliana na kauli ifuatayo: "Nyumbani kwetu, wanawake na wasichana ambao wako hedhini wanazuiliwa kufanya shughuli fulani."
     NDIO  HAPANA  Sijui

    Kama ulijibu NDIO, shughuli gani? Chagua zote zinazokubalika

    a)  Kushika vyanzo vya maji au wanyama
    b)  Kupika
    c)  Kufua
    d)  Mikutano ya hadhara kama vile mikutano ya kidini, mikutano ya jumuiya
    e)  Kulala sehemu yake ya kila siku
    f)  Kutumia choo ambacho huwa anatumia siku zote
    g)  Nyingine: ________________________________

14. Unakubali kwamba wasichana lazima waolewe mara tu baada ya kuvunja ungo.
     nakubaliana sana  nakubaliana  katikati  sikubaliani  sikubaliani kabisa

15. Unakubali kawmba wasichana wanakuwa wakubwa kiasi cha kutosha kuanza kufanya mapenzi wakishavunja ungo
     nakubaliana sana  nakubaliana  katikati  sikubaliani  sikubaliani kabisa

**Kipengele 7: Jibu sehemu hii kama tu umewahi kupata hedhi.**

1. Umewahi kupata hedhi?

     NDIO (endelea)  HAPANA (ruka hadi kipengele kinachofuata (Kipengele 7))

2. Wakati wa hedhi yako ya mwisho, je, kulikuwa na siku ambazo hukuweza kuja shuleni kwa sababu ya hedhi?
     NDIO  HAPANA
    Kama ndio ulibidi ukae nyumbani kwa siku ngapi kwa ajili ya hedhi? _____________

    Kama ndio, kwanini ulikosa kuja shule? Tafadhali chagua yote yanayokubalika.
     maumivu  kutokuwa na cha kutumia
     kuogopa nitavuja  kuogopa harufu
     kuona aibu  hakuna ba kubadilishia [vifaa vya usafi]
     kutoruhusiwa kushiriki  kusikia kutapika
     kutojisikia raha  nyingine ________

**SUPPLEMENTARY MATIERAL**

Surveying date:
School ID:
Classroom ID:
Student ID:

---

3. Wakati wa hedhi yako ya mwisho, uliwahi kuondoka shule mapema kwa sababu ya hedhi yako?
    NDIO  HAPANA
   Kama ndio, kwanini uliondoka mapema? Tafadhali chagua yote yanayokubalika.
    maumivu  nilichoka
    kuhitaji kubadilishia [vifaa vya usafi]  kuhitaji kunawa
    kuvuja  kutaniwa
    nyingine ____________________

4. Wakati wa hedhi yako ya mwisho, je, **ulishiriki** darasani kama ambavyo huwa ni kawaida yako kushiriki (kipindi hauko hedhini)?
    NDIO  HAPANA
   Kama ndio, kwanini haukushiriki sana? Tafadhali chagua yote yanayokubalika.
    woga  aibu
    maumivu ya tumbo na jumla  vigumu kuwa makini
    kuogopa kusimama  kuchoka
    nyingine ________

5. Wakati wa hedhi yako ya mwisho, uliweza kuwa makini darasani kama ambavyo huwa ni kawaida yako kushiriki (kipindi hauko hedhini)?
    NDIO  HAPANA
   Kama hapana, kwanini haukuweza kuwa makini kama kawaida? Tafadhali chagua yote yanayokubalika.
    woga  aibu
    maumivu ya tumbo na jumla  vigumu kuwa makini
    kuogopa kusimama  kuchoka
    nyingine ________

6. Je, unaogopa kutaniwa kuhusu kuvuja damu ya hedhi shuleni?
    NDIO  HAPANA

   Kama ndio, na nani? Tafadhali chagua yote yanayokubalika.
    wasichana wa rika lako  wavulana wa rika lako  walimu
    hakuna mtu  wengine ______

7. Unaogopa kutaniwa kuhusu harufu shuleni ukiwa hedhini?
    NDIO  HAPANA

   Kama ndio, nan ani? Tafadhali chagua yote yanayokubalika.
    wasichana wa rika lako  wavulana wa rika lako  walimu
    hakuna mtu  wengine ______

8. Unaogopa kujibu maswali darasani ukiwa hedhini?
    NDIO  HAPANA

9. Unafikiri baadhi ya wanafunzi wenzako wanaweza kutambua ukiwa hedhini?
    Ndio, mara nyingi  Ndio, mara chache  Hapana, kamwe

Surveying date:
School ID:
Classroom ID:
Student ID:

---

**Kipengele 8: Kutaniwa**

1. Je, umewahi kutaniwa kuhusu hedhi yako?
   □ NDIO □ HAPANA
2. Kutoka kwa wale marafiki wa KIUME uliowaandika juu (Kipengele 2, Swali 2), **wangapi** umewaona wakiwatania wasichana kuhusu hedhi zao?
   □ Hakuna □ Rafiki 1 □ Marafiki 2 □ Marafiki 3 □ Marafiki 4 □ Marafiki 5
3. Kutoka kwa wale marafiki wa KIKE uliowaandika juu (Kipengele 2, Swali 1), umeshawahi kujadili kuhusu hedhi ni **wangapi** kati yao?
   □ Hakuna □ Rafiki 1 □ Marafiki 2 □ Marafiki 3 □ Marafiki 4 □ Marafiki 5
4. Je, ni sawa kwa msichana kumuomba mpenzi wake kulipia bidhaa?
   □ NDIO □ HAPANA
   Kama ndio, zipi?
   ______________________________________________

**Kipengele 9: Maswali juu ya Vitu Tunavyovijali**

1. Walimu wangu hunitia moyo na kunipa msaada
   □ nakubali sana □ nakubali □ katikati □ sikubali □ sikubali sana
2. Elimu ni ya muhimu zaidi kwa wavulana kuliko wasichana
   □ nakubali sana □ nakubali □ katikati □ sikubali □ sikubali sana
3. Nina wasiwasi karibu muda wote
   □ nakubali sana □ nakubali □ katikati □ sikubali □ sikubali sana
4. Nimeridhika na Maisha yangu
   □ nakubali sana □ nakubali □ katikati □ sikubali □ sikubali sana

**Kama hujapata hedhi, nenda Kipengele 10.**

1. Najisikia aibu nikiwa hedhini
   □ nakubali sana □ nakubali □ katikati □ sikubali □ sikubali sana
2. Najisikia mwenye afya nikipata hedhi
   □ nakubali sana □ nakubali □ katikati □ sikubali □ sikubali sana
3. Najisikia kutengwa kutoka wengine nikiwa hedhini
   □ nakubali sana □ nakubali □ katikati □ sikubali □ sikubali sana
4. Najisikia wasiwasi nikiwa hedhini
   □ nakubali sana □ nakubali □ katikati □ sikubali □ sikubali sana

Surveying date:
School ID:
Classroom ID:
Student ID:

---

5. Naogopa kwamba nanuka nikiwa hedhini
   ☐ nakubali sana ☐ nakubali ☐ katikati ☐ sikubali ☐ sikubali sana

6. Najisikia jasiri zaidi nikitumia pedi nikiwa hedhini
   ☐ nakubali sana ☐ nakubali ☐ katikati ☐ sikubali ☐ sikubali sana

**Kipengele 10: Maswali ya Bei na WTP**

1. Tutakuuliza maswali kadhaa ambapo unaweza ukachagua kutoka vitu viwili. Tazama kila swali kama linajitegemea. Jibu kila swali kiukweli kwa sababu unaweza kujishindia chaguo lako.
   a. Ungependelea …?
      ☐ Pedi ya kutumia zaidi ya mara moja AU ☐ TSH 0

   b. Ungependelea …?
      ☐ Pedi ya kutumia zaidi ya mara moja AU ☐ TSH 1500

   c. Ungependelea …?
      ☐ Pedi ya kutumia zaidi ya mara moja AU ☐ TSH 3000

   d. Ungependelea …?
      ☐ Pedi ya kutumia zaidi ya mara moja AU ☐ TSH 4500

   e. Ungependelea …?
      ☐ Pedi ya kutumia zaidi ya mara moja AU ☐ TSH 6000

   f. Ungependelea …?
      ☐ Pedi ya kutumia zaidi ya mara moja AU ☐ TSH 7500

   g. Ungependelea …?
      ☐ Pedi ya kutumia zaidi ya mara moja AU ☐ TSH 9000

   h. Ungependelea …?
      ☐ Pedi ya kutumia zaidi ya mara moja AU ☐ TSH 10,000

   i. Ungependelea …?
      ☐ Pedi ya kutumia zaidi ya mara moja AU ☐ TSH 11,500

   j. Ungependelea …?
      ☐ Pedi ya kutumia zaidi ya mara moja AU ☐ TSH 13,000

**Kipengele 11: Demografia**

1. Jina lako nani? (la kwanza na la pili)________________________________________

2. Umeishi muda gani katika makazi uliyopo? _______________________

**SUPPLEMENTARY MATIERAL**

Surveying date:
School ID:
Classroom ID:
Student ID:

---

3. Umezaliwa mwezi gani na mwaka gani?________________________________________

4. Ulifikisha miaka mingapi kwenye tarehe yako ya kuzaliwa iliyopita? ________________________________________

5. Upo darasa la ngapi?__________________________________________________

6. Ulianza shul ukiwa na umri gani? ___________________________________________

7. Tafadhali chagua kabila lako linalokutambulisha
   - ☐ Mchagga
   - ☐ Mmasai
   - ☐ Msukuma
   - ☐ Mhaya
   - ☐ Msumbwa
   - ☐ kabila linginelo:_________________________________

8. Tafadhali chagua dhehebu linalokutambulisha
   - ☐ Mkristo
   - ☐ Muislamu
   - ☐ Dhehebu jinginelo:_________________________________
   - ☐ Sina dhehebu

9. Unaishi na nani kwa sasa?

| | | |
|---|---|---|
| i. ☐ wazazi | ☐ bibi na babu | ☐ peke yako |
| ii. ☐ ndugu uliozaliwa nao | ☐ wanafamilia wengine | ☐ bweni la shule |
| iii. ☐ bweni/hosteli binafsi | ☐ nyingine ______________________ | |

10. Unaweza kuoga nyumbani?

    iv. ☐ NDIO ☐ HAPANA

11. Unatumia jumla ya kiasi gani cha pesa kwa hedhi? ___________

12. Chanzo chako kikuu cha pesa ni kipi? Tafadhali chagua moja.

| | | |
|---|---|---|
| ☐ mama | ☐ baba | ☐ mpenzi |
| ☐ dada / kaka | ☐ nyingine | ☐ kazi |
| ☐ ufadhili | | |

13. Huwa unaitumia [hela] kufanya nini? Tafadhali chagua matumizi yako makuu matatu.
    - ☐ nguo
    - ☐ usafi binafsi
    - ☐ vipodozi
    - ☐ simu ya mkononi
    - ☐ pedi
    - ☐ nywele
    - ☐ vifaa vya shule
    - ☐ kitabu

---

 usafiri
 nyingine

14. Unatumia kiasi gani cha pesa kwenye pedi kila hedhi? ____________________________

15. Umekosa shule ndani ya siku 7 zilizopita?
 NDIO  HAPANA

Kama Ndio, sababu ilikuwa nini? ____________________________________________________

Surveying date:
School ID:
Classroom ID:
Student ID:

# GIRLS QUESTIONNAIRE

**Questionnaire**

**Section 1: Friends**

1. We would now like you to tell us about the 5 closest **FEMALE** friends you have in the school. Please mention the names of your closest friends. Only list friends who go to this school, and who are enrolled in the same year as you, and that you count as friends.

*Write the full name and class of the friends mentioned*

**a.** ___________________________________
**b.** ___________________________________
**c.** ___________________________________
**d.** ___________________________________
**e.** ___________________________________

2. We would now like you to tell us about the 5 closest **MALE** friends you have in the school. Please mention the names of your closest friends. Only list friends who go to this school, and who are enrolled in the same year as you, and that you count as friends.

*Write the full name and class of the friends mentioned*

**a.** ___________________________________
**b.** ___________________________________
**c.** ___________________________________
**d.** ___________________________________
**e.** ___________________________________

**Section 2: Information**
*We would now like to ask you some questions about periods.*

1. Why do a girl or a woman get her period?. Select all that apply.
   - ☐ personal sin
   - ☐ due to outside evil forces
   - ☐ because she did not become pregnant that month
   - ☐ release of bad blood
   - ☐ other
   - ☐ I do not know

2. Approximately, how often does a girl (who have ever had her period) get her period? Select one.
   - ☐ once in her life
   - ☐ once every year
   - ☐ every week
   - ☐ every month

Surveying date:
School ID:
Classroom ID:
Student ID:

---

 I do not know

3. How long does a menstrual period last on average? Select one.
    one hour
    one day
    a few days
    two weeks
    all the time
    I do not know

4. At what age do girls generally get their period for the first time?

    ______________________________________________

5. At what age do women stop getting their period?

    ______________________________________________

*6.* Select the correct answer
    *Please select Yes or No to indicate if the following statements are true.*

    h) When a girl reaches menarches (her first menstruation), it means she could get pregnant if she has sex.
     Yes  No  I do not know
    i) Menstruation blood is the shedding of the endometrium lining
     Yes  No  I do not know
    j) Menstrual period indicates a woman is not pregnant
     Yes  No  I do not know
    k) Ovulation happens on average 14 days before period
     Yes  No  I do not know
    l) Ovulation happens during period
     Yes  No  I do not know
    m) Is it common for girls to have physical discomfort when they have their period?
     Yes  No  I do not know
    n) Do most girls experience the following on their period?
    Bad mood  Yes  No  I do not know
    Stress  Yes  No  I do not know
    Fatigue  Yes  No  I do not know

**Section 3: Personal Experience**

1. Has anyone told you about girls' periods and why it is happening?
     YES  NO

    If so, who? Please select all that apply.
     mother  father
     grandmother  grandfather

**SUPPLEMENTARY MATIERAL**

Surveying date:
School ID:
Classroom ID:
Student ID:

---

| | |
|---|---|
| ☐ sister | ☐ brother |
| ☐ female friend | ☐ male friend |
| ☐ aunt or other female family member | ☐ uncle or male family member |
| ☐ health worker | ☐ priest / religious leader |
| ☐ female teacher | ☐ male teacher |
| ☐ boyfriend/partner | ☐ other |

2. Did you ever receive information about menstruation from the following:

| | | | |
|---|---|---|---|
| a. | Internet | ☐ YES | ☐ NO |
| b. | Health worker | ☐ YES | ☐ NO |
| c. | School materials (books, classes, homework, etc.) | ☐ YES | ☐ NO |
| d. | Informational pamphlet (ads, billboards) | ☐ YES | ☐ NO |

3. What was your reaction the first time that you learned about periods? Were you…
(Please select one from each group 5, where 3 is neutral.)

| | **1** | **2** | **3** | **4** | **5** | |
|---|---|---|---|---|---|---|
| surprised | ☐ | ☐ | ☐ | ☐ | ☐ | not at all surprised |
| afraid | ☐ | ☐ | ☐ | ☐ | ☐ | not at all afraid |
| excited | ☐ | ☐ | ☐ | ☐ | ☐ | not at all excited |
| confused | ☐ | ☐ | ☐ | ☐ | ☐ | not at all confused |
| confident | ☐ | ☐ | ☐ | ☐ | ☐ | not at all confident |
| disgusted | ☐ | ☐ | ☐ | ☐ | ☐ | not at all disgusted |
| proud | ☐ | ☐ | ☐ | ☐ | ☐ | not at all proud |
| ashamed | ☐ | ☐ | ☐ | ☐ | ☐ | not at all ashamed |

4. Has anyone told you what you should do when you start menstruating?
☐ YES ☐ NO

If so, what advice did you receive? Please select all that apply.
☐ how to use towels/fabrics/linen
☐ how to stay clean/wash
☐ how to manage pain
☐ things/activities to avoid during menstruation

Surveying date:
School ID:
Classroom ID:
Student ID:

---

☐ other ___________

5. Check all the people that you would **WANT** to talk to about your period.

| | |
|---|---|
| ☐ mother | ☐ father |
| ☐ grandmother | ☐ grandfather |
| ☐ sister | ☐ brother |
| ☐ female friend | ☐ male friend |
| ☐ aunt or other female family member | ☐ uncle or male family member |
| ☐ health worker | ☐ priest / religious leader |
| ☐ female teacher | ☐ male teacher |
| ☐ boyfriend/partner | ☐ other |
| ☐ no one | |

6. With whom would you **ABSOLUTELY NOT** want to talk to about your period. Please select all that apply.

| | |
|---|---|
| ☐ mother | ☐ father |
| ☐ grandmother | ☐ grandfather |
| ☐ sister | ☐ brother |
| ☐ female friend | ☐ male friend |
| ☐ aunt or other female family member | ☐ uncle or male family member |
| ☐ health worker | ☐ priest / religious leader |
| ☐ female teacher | ☐ male teacher |
| ☐ boyfriend/partner | ☐ other |
| ☐ no one | |

7. Have you had your period?
   ☐ Yes **(continue to next question)** ☐ No **(go to Section 4, Question 1)**

8. At what age did you have your first period? _______

9. Was the first time that you got your period a positive, negative, or neutral experience?
   ☐ positive ☐ negative ☐ neutral

10. What was your reaction the first time that you got your period? Were you…
(Please select one from each group 5, where 3 is neutral.)

| | **1** | **2** | **3** | |
|---|---|---|---|---|
| 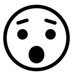 surprised | ☐ | ☐ | ☐ | not at all surprised |
| 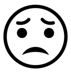 afraid | ☐ | ☐ | ☐ | not at all afraid |
| 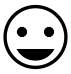 excited | ☐ | ☐ | ☐ | not at all excited |

| | | | | |
|---|---|---|---|---|
| confused | ☐ | ☐ | ☐ | not at all confused |
| confident | ☐ | ☐ | ☐ | not at all confident |
| disgusted | ☐ | ☐ | ☐ | not at all disgusted |
| proud | ☐ | ☐ | ☐ | not at all proud |
| ashamed | ☐ | ☐ | ☐ | not at all ashamed |

11. Think back to the time before you got your period. Did anyone tell you about your period before it started?

☐ YES ☐ NO

If so, who? Please select all that apply.

If so, who? Please select all that apply.

| | |
|---|---|
| ☐ mother | ☐ father |
| ☐ grandmother | ☐ grandfather |
| ☐ sister | ☐ brother |
| ☐ female friend | ☐ male friend |
| ☐ aunt or other female family member | ☐ uncle or male family member |
| ☐ health worker | ☐ priest / religious leader |
| ☐ female teacher | ☐ male teacher |
| ☐ boyfriend/partner | ☐ other |

12. How many days does your period last?

__________days

13. From a scale of 1 to 5 where 1 is no pain and 5 very strong pain; How much physical pain do you experience when you have your period?

| | **1** | **2** | **3** | **4** | **5** | |
|---|---|---|---|---|---|---|
| *No pain at all* | ☐ | ☐ | ☐ | ☐ | ☐ | *Very strong pain* |

14. On a scale from 1 to 5, where 1 is less pain and 5 is more pain and 3 is the same; How do you think that the pain you feel during your period is compared to the pain that your peers experience?

| | **1** | **2** | **3** | **4** | **5** | |
|---|---|---|---|---|---|---|
| *I experience much less pain* | ☐ | ☐ | ☐ | ☐ | ☐ | *I experience much stronger pain* |

15. From a scale of 1 to 5 where 1 is very light bleeding and 5 very heavy bleeding; How much do you bleed when you have your period?

| | **1** | **2** | **3** | **4** | **5** | |
|---|---|---|---|---|---|---|
| *very light bleeding* | ☐ | ☐ | ☐ | ☐ | ☐ | *Very heavy bleeding* |

Surveying date:
School ID:
Classroom ID:
Student ID:

---

16. On a scale from 1 to 5, where 1 is lighter bleeding and 5 is heavier bleeding and 3 is the same; Do you think that you bleed more, less, or the same as your peers?

| | **1** | **2** | **3** | **4** | **5** | |
|---|---|---|---|---|---|---|
| *I have much lighter bleeding* | ☐ | ☐ | ☐ | ☐ | ☐ | *I have much heavier bleeding* |

17. A menstrual cycle lasts from the first day of period (bleeding) until the day before the beginning of the next period (first day of bleeding). Do you know how long your menstrual cycle is on average? In other word, do you keep track of how often do you bleed?
    ☐ YES ☐ NO

18. Do you think that for you the time between bleedings varies a lot?
    ☐ YES ☐ NO

19. Do you find it hard to know when you are going to bleed soon and be prepared?
    ☐ YES ☐ NO

20. During your period, or immediately before, do you ever have…:

| | | |
|---|---|---|
| Bad mood? | ☐ YES | ☐ NO |
| Stress or anxiety? | ☐ YES | ☐ NO |
| Fatigue? | ☐ YES | ☐ NO |

21. Do you use painkillers (like for example aspirin) to manage pain when you have your period?
    ☐ Always ☐ Sometimes ☐ Rarely ☐ Never

**BREAK HERE FOR SECTION 4 WITH AFRIPADS**

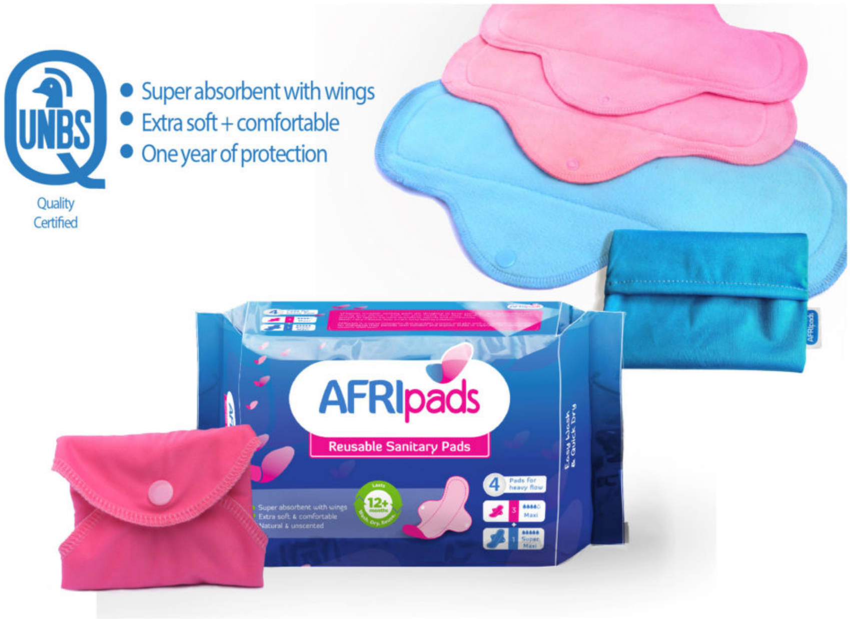


*Pedi za kusafisha zinazotumika zaidi ya mara moja ni kitambaa kinachovaliwa kwenye chupi (nguo ya ndani) kuzuia damu ya hedhi kuvujia kwenye nguo. Inaweza kuoshwa, kukaushwa na kutumiwa kwa mwaka 1. Picha hapo juu inaonesha Afripads, aina mojawapo ya pedi za kutumiwa zaidi ya mara moja. Kila pakiti ina pedi 4.*

**SUPPLEMENTARY MATIERAL**

Surveying date:
School ID:
Classroom ID:
Student ID:

---

1. *Je umewahi Kuona ama Kusikia kuhusu pedi za kusafisha zinazotumika Zaidi ya mara moja?*

   ☐ *Ndio* ☐ *Hapana*

2. *Je umewahi Kutumia pedi za kusafisha zinazotumika Zaidi ya mara moja?*

   ☐ *Ndio* ☐ *Hapana*

3. What do you **sometimes** use when you are menstruating? Please select **all that apply.**
   ☐ cloth ☐ pads
   ☐ tampons ☐ natural materials (grass, leaves, cow dung, etc.)
   ☐ mattress stuffing ☐ menstrual cup
   ☐ nothing ☐ other _________

4. What do you **most often** use when you are menstruating? Please select **only one**.
   ☐ cloth ☐ pads
   ☐ tampons ☐ natural materials (grass, leaves, cow dung, etc.)
   ☐ mattress stuffing ☐ menstrual cup
   ☐ nothing ☐ other _________

5. Do you ever use cloth?
   ☐ YES ☐ NO
   a. If you ever use a cloth, do you wash the fabrics you use during menstruation with soap?
   ☐ YES ☐ NO ☐ I don't use a cloth
   b. If you ever use a cloth, do you dry the fabrics you have washed in the sun?
   ☐ YES ☐ NO ☐ I don't use a cloth
   If no, where ________________________________________

**6.** What is your preferred method of menstrual sanitation? Please **select one.**
   ☐ cloth ☐ pads
   ☐ tampons ☐ natural products (grass, leaves, cow dung, etc.)
   ☐ mattress stuffing ☐ menstrual cup
   ☐ nothing ☐ other __________________________________

7. Do you always use your preferred method?
   ☐ YES ☐ NO

8. Think of a month when you did not use your preferred method. Why did you **NOT** use your preferred method?
   ☐ not enough money to buy ☐ the method wasn't available
   ☐ no parental permission ☐ I didn't know how to
   ☐ I was embarrassed ☐ the method wasn't safe
   ☐ other ______________________________

9. How often do you change sanitation during your period each day? __________________

Surveying date:
School ID:
Classroom ID:
Student ID:

---

10. How do you dispose of your sanitary product (**the one that you chose in question 24**)?

☐ I don't (I use it over and over) ☐ dust bin
☐ toilet/latrine ☐ burn it
☐ bury it ☐ other ___________________________

**Section 4: Peers' Period Management (UPDATED TO SECTION 5)**

1. Please estimate the share of female classmates who have received their periods.

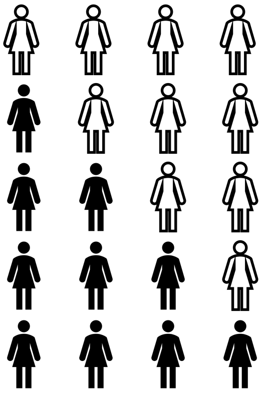

☐ 0%
☐ 1-25%
☐ 26-50%
☐ 51-75%
☐ 76-100%

2. Sometimes girls are teased about their periods. Please estimate the share of female classmates who have been teased about their periods. .

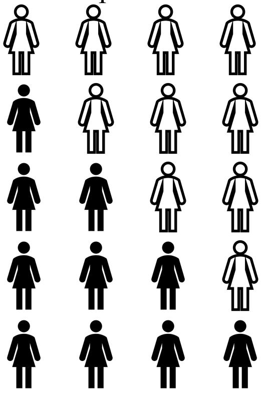

☐ 0%
☐ 1-25%
☐ 26-50%
☐ 51-75%
☐ 76-100%

3. Please estimate **the most common** method of menstrual sanitation among your peers in the class. Please select only one.

☐ cloth ☐ pads
☐ tampons ☐ natural materials (grass, leaves, cow dung, etc.)
☐ mattress stuffing ☐ menstrual cup
☐ nothing ☐ other _________

4. How confident are you that you know what your female classmates use when they are menstruating?

*Not at all confident* ☐1 ☐2 ☐3 ☐4 ☐5 *Very confident*

Surveying date:
School ID:
Classroom ID:
Student ID:

---

5. If they use sanitary pads, do you know how your female peers dispose of them?
 YES  NO

6. If you answered yes to the question above, what is the most common thing they do?
 They don’t dispose of them (they use it over and over)
 dust bin
 toilet/latrine
 burn them
 bury them
 other ____________________________________________________

**Section 6: Management at School**

1. Do you feel safe in the latrines at school?
 YES  NO

2. Do you feel safe in the latrines at school when you have your period?
 YES  NO

3. Do you know when your female classmates have their period?
 YES  NO

4. Do you feel like having period is something that one must hide from others?
 YES  NO

5. Would you feel ashamed if other girls knew that you were on your period?
 YES  NO

6. Would you feel ashamed if boys knew that you were on your period?
 YES  NO

7. If people knew that you have ever received your period, would you be afraid of teasing?
 YES  NO
If so, by whom? Please select all that apply.
 female peers  male peers  teachers  no one  other______

8. If people knew that you have ever received your period, would you be afraid of being touched against your will, or asked insistently to go out with someone?
 YES  NO
If so, by whom? Please select all that apply.
 peers  teachers  family members  no one  other______

If so, where are you afraid that this would occur?
 public bathrooms
 school bathrooms
 class room
 school yard

---

 on my way to or from school
 public space (such as a square)
 home
 nowhere
 other ______________________

9. If people knew that you have your period, would you be afraid of unwanted pregnancies or dishonor?
 YES  NO

10. If people knew that you have ever received your period, would you be afraid of teachers not being understanding or helpful?
 YES  NO

11. If people knew that you have ever received your period, would you afraid of the pressure to marry or take a boyfriend?
 YES  NO

12. Do you agree with the following statement: "In my house, women/girls should be ashamed to talk about or reveal their period (blood, pains, sanitary materials) in front of my family members."
 strongly agree  agree  neutral  disagree  strongly disagree

13. Do you agree with the following statement: "In my house, women and girls who are menstruating are restricted from certain activities."
 YES  NO  Don't know

If you answered YES, what activities? Select all that apply

h)  Touching water sources or animals
i)  Cooking
j)  Washing dishes
k)  Public gatherings like religious gatherings, community meetings
l)  Sleeping in her normal spot
m)  Using the normal toilet/latrine
n)  Other: ____________________________

14. Do you agree that girls should get married as soon as they get their period.
 strongly agree  agree  neutral  disagree  strongly disagree

15. Do you agree that girls are old enough to have sex after they get their period
 strongly agree  agree  neutral  disagree  strongly disagree

**Section 7: Only fill this in if you ever had your period.**

1. Did you ever have a period?

Surveying date:
School ID:
Classroom ID:
Student ID:

---

☐ YES (continue) ☐ NO (skip to next section (Section 7)) (NOW SECTION 8)

2. During your last period, were there days when you could not come to school because of your period?
   ☐ YES ☐ NO
   If yes how many days did you have to stay at home because of your period? _____________

   If yes, why did you miss school? Please select all that apply.
   ☐ pain ☐ nothing to use
   ☐ afraid I will leak ☐ afraid of odor
   ☐ ashamed ☐ no place to change
   ☐ not allowed to take part ☐ feel sick
   ☐ don't feel comfortable ☐ other ________

3. During your last period, did you ever leave school early because of your period?
   ☐ YES ☐ NO
   If yes, why did you leave early? Please select all that apply.
   ☐ pain ☐ tired
   ☐ needed to change ☐ needed to wash
   ☐ leaked ☐ teased
   ☐ other ______________________________________________________

4. During your last period, did you **participate** as much in class as you normally do (when you do not have your period)?
   ☐ YES ☐ NO
   If no, why did you not participate as much? Please select all that apply.
   ☐ fear ☐ shame
   ☐ cramps and pain ☐ hard to concentrate
   ☐ afraid to stand up ☐ tired
   ☐ other _______________

5. During your last period, did you **concentrate** as much in class as you normally do (when you do not have your period)?
   ☐ YES ☐ NO
   If no, why did you not concentrate as much? Please select all that apply.
   ☐ fear ☐ shame
   ☐ cramps and pain ☐ hard to concentrate
   ☐ afraid to stand up ☐ tired
   ☐ other _______________

6. Are you afraid of being teased about leaking menstrual blood at school?
   ☐ YES ☐ NO

   If so, by whom? Please select all that apply.
   ☐ female peers ☐ male peers ☐ teachers ☐ no one ☐ other______

7. Are you afraid of being teased about odor at school when you have your period?

Surveying date:
School ID:
Classroom ID:
Student ID:

---

 YES  NO

If so, by whom? Please select all that apply.
 female peers  male peers  teachers  no one  other______

8. Are you afraid of answering a question in the classroom when you have your period?
 YES  NO

9. Do you think that some of your classmates can tell when you have your period?
 Yes, most of the time  Yes, only sometimes  No, never

**Section 8: Teasing**

1. Have you ever been teased about your period?
 YES  NO

2. From the MALE friends you wrote above (Section 2, Question 2), **how many** have you seen teasing girls about their periods?
 No friend  1 friend  2 friends  3 friends  4 friends  5 friends

3. From the FEMALE friends you wrote above (Section 2, Question 1), with **how many** have you ever discussed periods?
 No friend  1 friend  2 friends  3 friends  4 friends  5 friends

4. Is it okay for a girl to ask her boyfriend to pay for items?
 YES  NO
If so, what?
____________________________________________________________

**Section 9: Value Questions**

1. My parents/caretakers encourage me to do well in school
 strongly agree  agree  neutral  disagree  strongly disagree

2. My teachers give me a lot of encouragement and support
 strongly agree  agree  neutral  disagree  strongly disagree

3. Education is more important for boys than for girls
 strongly agree  agree  neutral  disagree  strongly disagree

4. Things in the future will work out OK for me
 strongly agree  agree  neutral  disagree  strongly disagree

5. I am worried nearly all the time
 strongly agree  agree  neutral  disagree  strongly disagree

Surveying date:
School ID:
Classroom ID:
Student ID:

---

6. I am satisfied with my life
   ▯ strongly agree ▯ agree ▯ neutral ▯ disagree ▯ strongly disagree

**If you have NOT had your period, go to Section 9.**

7. I feel ashamed during my period
   ▯ strongly agree ▯ agree ▯ neutral ▯ disagree ▯ strongly disagree

8. I feel healthy when I have my period
   ▯ strongly agree ▯ agree ▯ neutral ▯ disagree ▯ strongly disagree

9. I feel isolated from others during my period
   ▯ strongly agree ▯ agree ▯ neutral ▯ disagree ▯ strongly disagree

10. I feel worried during my period
   ▯ strongly agree ▯ agree ▯ neutral ▯ disagree ▯ strongly disagree

11. I worry that I smell when I have my period
   ▯ strongly agree ▯ agree ▯ neutral ▯ disagree ▯ strongly disagree

12. I feel more confident during my period when using pads
   ▯ strongly agree ▯ agree ▯ neutral ▯ disagree ▯ strongly disagree

**Section 10: Price Questions and WTP**

1. We will ask you a few questions where you can choose between two items. Treat each question as a separate question. Answer each question truthfully because you might win your choice.
   a. Would you prefer…?
      ▯ A reusable sanitary pad OR ▯ TSH 0

   b. Would you prefer…?
      ▯ A reusable sanitary pad OR ▯ TSH 1500

   c. Would you prefer…?
      ▯ A reusable sanitary pad OR ▯ TSH 3000

   d. Would you prefer…?
      ▯ A reusable sanitary pad OR ▯ TSH 4500

   e. Would you prefer…?
      ▯ A reusable sanitary pad OR ▯ TSH 6000

   f. Would you prefer…?
      ▯ A reusable sanitary pad OR ▯ TSH 7500

   g. Would you prefer…?
      ▯ A reusable sanitary pad OR ▯ TSH 9000

Surveying date:
School ID:
Classroom ID:
Student ID:

---

h. Would you prefer…?
 A reusable sanitary pad OR  TSH 10,000

i. Would you prefer…?
 A reusable sanitary pad OR  TSH 11,500

j. Would you prefer…?
 A reusable sanitary pad OR  TSH 13,000

**Section 11: Demographics**

1. What is your name? (first and last) ______________________________

2. How long have you been living continuously in (place of residence)? ______________________

3. In what month and year where you born?______________________________

4. How old were you at your last birthday? ______________________________

5. What grade are you in?______________________________

6. At what age did you start school? ______________________________

7. Please select the ethnicity that best describes you:

 Chagga
 Masai
 Sukuma
 Wahaya
 Wasumbwa
 Other :______________________________

8. Please select the religion that best describes you:

 Christian
 Muslim
 Chagga
 Other :______________________________
 No Religion

9. Who do you currently live with?
   i.  parents  grandparents  alone
   ii.  siblings  other family members  boarding school
   iii.  boarding house/hostel  other
   ______________________________

10. Can you wash your body at home?
   i.  YES  NO

**SUPPLEMENTARY MATIERAL**

Surveying date:
School ID:
Classroom ID:
Student ID:

---

11. How much money do you spend per month total? ___________

12. What is your main source of money? Please select one.
    - ☐ mother
    - ☐ father
    - ☐ girlfriend/boyfriend
    - ☐ sister /brother
    - ☐ other
    - ☐ work
    - ☐ scholarship

13. What do you spend it on? Please select the top three expenses.
    - ☐ clothes
    - ☐ personal hygiene
    - ☐ make up
    - ☐ mobile phone
    - ☐ sanitary pads
    - ☐ hair
    - ☐ school products
    - ☐ food
    - ☐ transport
    - ☐ other

14. How much do you spend on sanitary pads per month? ___________________________

15. Did you miss school in the past 7 days?
    ☐ YES ☐ NO

If Yes, what was the reason? ___________________________________________________
