# Supplementary material for: Biological, material and socio-cultural constraints to effective menstrual hygiene management among secondary school students in Tanzania
Source: PLOS Glob Public Health. 2022 Mar 14;2(3):e0000110. doi: 10.1371/journal.pgph.0000110 (PMC10021794; doi:10.1371/journal.pgph.0000110)
Supplement: S2 File — (PDF) [file pgph.0000110.s005.pdf]

Social Constraints:
This value attempts to measure the degree of social constraints that the girls are exposed to. There is a total of 10 possible points, a higher score indicates that girls are facing more social constraints.

**Question Number:**<br>**& Number Missing**	**Question:**	**Answer Options:**	**Scoring:**
Section 6 Question 7 Part A<br><br>Missing: 33	If people knew that you have ever received your period, would you be afraid of teasing?	-Yes<br>-No	1 point will be given to students who selected "yes".
Section 6 Question 8 Part A<br><br>Missing: 53	If people knew that you have ever received your period, would be afraid of being touched against your will, or asked insistently to go out with someone?	-Yes<br>-No	1 point will be given to students who selected "yes".
Section 6 Question 9<br><br>Missing: 23	If people knew that you have your period, would be afraid of unwanted pregnancies or dishonor?	-Yes<br>-No	1 point will be given to students who selected "yes".
Section 6 Question 11<br><br>Missing: 47	If people knew that you have ever received your period, would be afraid of the pressure to marry or take a boyfriend?	-Yes<br>-No	1 point will be given to students who selected "yes".

Biological Constraints:

This value attempts to measure the degree of biological constraints that the girls are exposed to. There is a total of 11 possible points, a higher score indicates that girls are facing more biological constraints.

**Question Number & Number Missing:**	**Question:**	**Answer Options:**	**Scoring:**
Section 3 Question 13<br><br>Missing: 76	From a scale of 1 to 5 where 1 is no pain and 5 is very pain; How much physical pain do you experience when you have your period?	1 = No Pain<br>2<br>3<br>4<br>5 = Very Strong Pain	1 (Score = 0 points)<br>2 (Score = 1 point)<br>3 (Score = 2 points)<br>4 (Score = 3 points)<br>5 (Score = 4 points)
Section 3 Question 15<br><br>Missing: 82	From a scale of 1 to 5 where 1 is light bleeding and 5 very heavy bleeding; how much do you bleed when you have your period?	1 = Very light bleeding<br>2<br>3<br>4<br>5 = Very heavy bleeding	1 (Score = 0 points)<br>2 (Score = 1 points)<br>3 (Score = 2 points)<br>4 (Score = 3 points)<br>5 (Score = 4 points)

Material Constraints:
This value attempts to measure the degree of material constraints that the girls are exposed to. There is a total of 10 possible points, a higher score indicates that girls are facing more material constraints.

**Question Number & Number Missing:**	**Question:**	**Answer Options:**	**Scoring:**
Section 4 Question 7<br><br>Missing: 52	Do you always use your preferred MHM method?	-Yes<br>-No	1 point will be given to students who selected "no".
Section 11 Question 10<br><br>Missing: 27	Can you wash your body at home?	-Yes<br>-No	1 point will be given to students who selected "no".

Informational Constraints:
This value attempts to measure the degree of informational constraints that the girls are exposed to. There is a total of 13 possible points, a higher score indicates that girls are facing more informational constraints.

**Question Number:**	**Question:**	**Answer Options:**	**Scoring:**
Section 3 Question 1<br><br>Missing: 11	Has anyone told you about girls' periods and why it is happening	Yes<br>No	Students will receive 1 point if they answered "no".
Section 3 Question 4<br><br>Missing: 14	Has anyone told you what you should do when you start menstruating?	Yes<br>No	Students will receive 1 point if they answered "no".
